# Establishment of a Cre-loxP System Based on a Leaky *LAC4* Promoter and an Unstable panARS Element in *Kluyveromyces marxianus*

**DOI:** 10.3390/microorganisms10061240

**Published:** 2022-06-17

**Authors:** Haiyan Ren, Anqi Yin, Pingping Wu, Huanyu Zhou, Jungang Zhou, Yao Yu, Hong Lu

**Affiliations:** 1 State Key Laboratory of Genetic Engineering, School of Life Sciences, Fudan University, Shanghai 200438, China; 20110700056@fudan.edu.cn (H.R.); 19210700126@fudan.edu.cn (A.Y.); 19110700028@fudan.edu.cn (P.W.); 21110700057@m.fudan.edu.cn (H.Z.); zhoujg@fudan.edu.cn (J.Z.); 2 Shanghai Engineering Research Center of Industrial Microorganisms, Shanghai 200438, China; 3 National Technology Innovation Center of Synthetic Biology, Tianjin 300308, China; 4 Shanghai Collaborative Innovation Center for Biomanufacturing Technology, Shanghai 200237, China

**Keywords:** Cre-loxP, *Kluyveromyces marxianus*, chromosomal rearrangement

## Abstract

The Cre-loxP system produces structural variations, such as deletion, duplication, inversion and translocation, at specific loci and induces chromosomal rearrangements in the genome. To achieve chromosomal rearrangements in *Kluyveromyces marxianus*, the positions and sequences of centromeres were identified in this species for the first time. Next, a Cre-loxP system was established in *K. marxianus*. In this system, the Cre recombinase was expressed from a leaky *LAC4* promoter in a plasmid to alleviate the cytotoxicity of Cre, and the unstable plasmid contained a panARS element to facilitate the clearance of the plasmid from the cells. By using *LAC4* as a reporter gene, the recombination frequencies between loxP sites or loxPsym sites were 99% and 73%, respectively. A *K. marxianus* strain containing 16 loxPsym sites in the genome was constructed. The recombination frequency of large-scale chromosomal rearrangements between 16 loxPsym sites was up to 38.9%. Our study provides valuable information and tools for studying chromosomal structures and functions in *K. marxianus*.

## 1. Introduction

The Cre-loxP system is a useful tool for site-specific genetic manipulation, which is widely used to achieve deletion, insertion, duplication, transposition, inversion or translocation in bacteria [1], yeasts [2], plants [3,4], insects [5] and mammals [6]. The Cre-loxP system consists of two components, including a pair of 34-bp loxP sites and the Cre recombinase of the P1 bacteriophage [7]. Cre catalyzes the recombination between loxP sites, and the outcome of the recombination depends on the location and orientation of the loxP sites [8]. The Cre-loxP system does not require additional co-factors such as ATP or topoisomerase for efficient recombination [9] and it can mediate recombination on many types of DNA, including linear, circular and superhelical DNA [10]. Therefore, the Cre-loxP system was successfully applied to a wide range of scenarios. For example, the system was used to remove selection markers for the recycling of markers, which facilitated the manipulation of target genes in multiple rounds [11]. The system was applied as a switch of gene activation and inactivation in transgenic animals [12]. Combinations of different Cre recombinases and multiple loxP site variants, such as symmetrical loxP (loxPsym), created multiple recombination events and expanded the application scope of this technique [13]. In the Synthetic Yeast Genome Project (Sc2.0 Project), ~4000 loxPsym sites were integrated into all sixteen synthetic chromosomes [14]. The SCRaMbLE (Synthetic Chromosome Rearrangement and Modification by loxP-mediated Evolution) technique was developed to produce numerous genome rearrangements in the synthetic genome of Sc2.0 and artificially accelerate the evolution of yeast strains by this technique [15].

A key to the application of the Cre-loxP system was to control the expression level of Cre recombinase. Constitutive overexpression of Cre leads to at least two problems. First, overexpression of Cre recombinase caused toxicity and inhibited the growth of certain hosts, including mammalian cells, *Schizosaccharomyces pombe* and *Kluyveromyces marxianus* [16,17,18]. In mammalian cells, the toxicity of Cre depended on the endonuclease activity of Cre recombinase, which might induce DNA damage, such as chromosomal aberrations and sister chromatid exchanges [16]. Second, persistent expression of Cre recombinase might cause repetitive recombination between loxP sites and lead to genomic instability. Therefore, inducible systems were commonly employed to control the expression of Cre recombinase [19]. For example, Cre was expressed by an inducible *GAL1* promoter in *Saccharomyces cerevisiae* [20], *Kluyveromyces lactis* [21] and *Saccharomyces boulardii* [22]. Cre recombinase was fused with the estrogen-binding domain (EBD) of the murine estrogen receptor and the Cre-EBD fusion protein was induced from the cytoplasm to the nucleus by adding β-estradiol into the medium [23,24]. Recently, the photoinduction of Cre recombinase was developed in various model organisms [25,26,27]. During the inducible expression or activation of Cre recombinase, the level of activated Cre in uninduced conditions needs to be kept low. Alternatively, transient expression of Cre recombinase can be achieved by an unstable vector [28,29]. In the end, to ensure genomic stability of host cells, the expression of Cre recombinase needs to be shut off by removing the inducer from the medium [30] or by removing the plasmid from cells [24]. 

*K. marxianus* is a natural thermotolerant yeast, which is a promising host for the production of cellulosic ethanol, chemicals and heterologous proteins [31,32,33,34]. In our previous study, the toxicity of Cre recombinase has been proved in *K. marxianus* [18]. It raised the demand for strict regulation of Cre expression in *K. marxianus*. Meanwhile, the potential of the Cre-loxP system in mediating various structural variations, including inversion, translocation and large-scale chromosomal rearrangements, was not yet explored in *K. marxianus*. In this study, a Cre-loxP system was established in *K. marxianus*. Cre was expressed by a leaky *LAC4* promoter in a plasmid to reduce its toxicity. The plasmid could be cleared from cells in the unselective medium rapidly, as the plasmid contained an exogeneous autonomously replicating sequence from *K*. *lactis* (panARS) [35]. The Cre-loxP was successfully applied in mediating deletion and inversion between loxP and loxPsym sites in *K. marxianus*, while the highest efficiency of deletion reached 99%. In an engineered *K. marxianus* strain containing 16 loxPsym sites across the genome, interchromosomal rearrangements were mediated by the Cre-loxP system with an efficiency of up to 38.9%. Our study provides valuable information and tools to study chromosomal rearrangements in *K. marxianus*.

## 2. Materials and Methods

### 2.1. Strains and Plasmids

Strains used in this study were listed in Supplementary File S1: Table S1. Plasmids used in this study were shown in Supplementary File S2: Table S2. Primers used in the construction of strains and plasmids were listed in Supplementary File S3: Table S3. Wild-type *K. marxianus* strain FIM-1 was deposited in the China General Microbiological Culture Collection Center (CGMCC No.10621). Wild-type *K. lactis* strain ATCC8585 was purchased from the ATCC. *URA3* was deleted in FIM-1 to obtain FIM-1∆U [32]. *HMR* locus was deleted in FIM-1∆U to obtain αFIM-1∆U and *HIS3* was deleted in αFIM-1∆U to obtain αFIM-1∆U∆H [36]. Genomic DNA was extracted by Yeast Genomic DNA Extraction Kit (D1900, Solarbio, Beijing, China).

Other strains used in this study were constructed with the aid of CRISPR plasmids and donor sequences. The CRISPR vector used in this study was LHZ531 [37]. Primers containing 20 bp target sequence were annealed in pairs and inserted into *Sap* I site of LHZ531 to obtain CRISPR plasmids LHZ891, LHZ897~LHZ913. The *TEF* promoter of *Ashbya gossypii* and the open reading frame (ORF) of *LAC4* flanked by two loxP sites were amplified by overlap PCR and ligated with pMD18-T to obtain LHZ894. The donor sequence was amplified from LHZ894 and co-transformed with CRISPR plasmid LHZ891 into αFIM-1∆U∆H to obtain LHP719. Similarly, *LAC4* flanked by two loxPsym sites was inserted into αFIM-1∆U∆H to obtain LHP756, or *LAC4* containing one loxPsym site upstream of its ORF was inserted into αFIM-1∆U∆H to obtain LHP758. A loxPsym site was inserted at 747,278 bp of chromosome I of LHP758 to obtain LHP1043. A total of 16 loxPsym sites was inserted into the genome of αFIM-1∆U to obtain LHP1044. Positions of 16 loxPsym sites were shown in Supplementary File S1: Table S1.

To construct plasmids used in the mitotic stability assay, *KANMX6* in the pFA6a-13Myc-KANMX6 [38] was replaced by *HphMX4* [39] by mutagenesis PCR and the vector backbone containing *HphMX4* was amplified from the resultant plasmid. The ARS1 and ARS1/*CEN5* were amplified from the genomic DNA of FIM-1, respectively. The *ADH1* promoter was amplified from the genomic DNA of S288C. ARS1 and *ADH1* promoter were ligated with the vector backbone by Gibson assembly to obtain LHZ881. ARS1/*CEN5* fragment and *ADH1* promoter were ligated with the vector backbone to obtain LHZ882. Sequences of LHZ881 and LHZ882 were provided in Supplementary Files S4 and S5. Centromere core sequences with flanking sequences were amplified from the genomic DNA of FIM-1 and then were inserted into *Sal* I site of LHZ881 vector to obtain a series of CEN plasmids LHZ883~LHZ890. 

LHP620 plasmid expressing Cre recombinase was described before [18]. ARS1 amplified from the genomic DNA of FIM1 and panARS amplified from the genomic DNA of ATCC8585 were inserted into *Pvu* II and *Sal* I sites of LHP620 to obtain LHZ892 and LHZ893, respectively. 

### 2.2. Media

*K. marxianus* and *K. lactis* cells were cultivated at 30 degrees. YPD medium (2% peptone, 2% glucose, 1% yeast extract, 2% agar for plates). SD medium (0.17% yeast nitrogen base without amino acids and ammonium sulfate (LA7420, Solarbio, Beijing, China), 2% glucose, 0.1% glutamic sodium, 2 g/L dropout mix, 2% agar for plates) was prepared as described before [40]. YPD or SD medium was supplemented with 0.2 g/L G418 (A600958, Sango), 200 mg/mL Hygromycin (H8080, Solarbio, Beijing, China) or 80 mg/L X-gal (A600083, Sangon, Shanghai, China) as indicated.

### 2.3. Mitotic Stability of CEN Plasmids

Mitotic stability assay was described as previously [41]. The LHZ881~LHZ890 were transformed into FIM-1∆U separately and selected on YPD + Hyg plates. Transformants were grown in 3 mL YPD + Hyg till stationary phase. The culture was diluted into 3 mL YPD to an initial OD_600_ of 0.1. After approximately 7 generations of unselective growth, the culture was diluted and spread onto YPD + Hyg and YPD plates. The percentage of cells containing the plasmid (N) was calculated as the ratio of the number of colonies on the YPD + Hyg plate to that on the YPD plate. The loss rate of plasmid per generation (X) was obtained using the formula:

X = (1 − e^((lnN7/N0)/7)^) × 100%

in which N_0_ and N_7_ were the percentages of cells containing the plasmid after 0 and 7 generations of unselective growth, respectively.

### 2.4. Recombination at LAC4 Locus

To induce recombination between loxP sites at the *LAC4* locus, LHZ620, LHZ892 and LHZ893 were transformed into LHP719 separately. Transformants were grown on SD + G418 + X-gal plates for 72 h. White and blue colonies on the plates were identified by colony PCR [42]. A PCR product of 751 bp using a primer pair YY402F/YY558R indicated the presence of *LAC4* at the original locus and a product of 720 bp using a primer pair YY402F/YY590R indicated the deletion of *LAC4*. To remove the Cre plasmid from the cells, transformants were scraped off the plates and suspended in sterile water. The mixture of the transformants was diluted into a YPD medium to start an OD_600_ of 0.2. Cells were grown for 2, 6, 12 and 24 h. At each timepoint, a portion of culture was diluted and spread onto SD + X-gal, YPD and YPD + G418 plates. The frequency of recombination at the *LAC4* locus was indicated by the percentage of white colonies on SD + X-gal plates. The percentage of cells that lost the plasmid was calculated as one minus the ratio of the number of colonies on the YPD + G418 plate to that on the YPD plate. To induce recombination between loxPsym sites at the *LAC4* locus, LHZ893 was transformed into LHP756. Transformants were selected in SD + G418 + X-gal plates, grown in YPD medium for 12 h and then spread onto SD + X-gal plates, as described above. White colonies were subjected to colony PCR. A PCR product of 1117 bp using primers YY402F/YY551F indicated the inversion of *LAC4*. To induce the translocation between loxPsym sites in different chromosomes, LHZ893 was transformed into LHP1043. Transformants were selected in SD + G418 + X-gal plates, grown in YPD medium for 12 h and then spread onto SD + X-gal plates. White colonies were subjected to colony PCR. A PCR product produced by R1 and R2 primers indicated the translocation between two chromosomes.

### 2.5. Recombination between 16 loxPsym Sites

LHZ893 was transformed into LHP1044 and transformants were grown on the YPD + G418 plate for 72 h. Transformants were scraped off the plate and suspended in sterile water. The mixture of the transformants was diluted into a YPD medium to start an OD_600_ of 0.2. Cells were grown for 12 h. The culture was diluted and spread onto YPD plates. Alternatively, to increase the frequency of recombination, transformants were mixed, spread onto YPD + G418 and grown for another 48 h. The process was repeated once to achieve a total of 96 h extra growth on the YPD + G418 plate. Then, cells were grown in the YPD medium for 12 h and spread onto the YPD plate as described above. Colonies on YPD plates were subjected to colony PCR using a series of primer pairs designed for each loxPsym site. The failure to obtain the expected product by using a primer pair suggested the rearrangements at the corresponding site. To confirm rearrangements, PCR was repeated by using the genomic DNA extracted from candidate colonies as the template. The primers were listed in Supplementary File S3: Table S3. 

## 3. Results

### 3.1. Identification of Centromeres of K. marxianus

**Figure 1:**
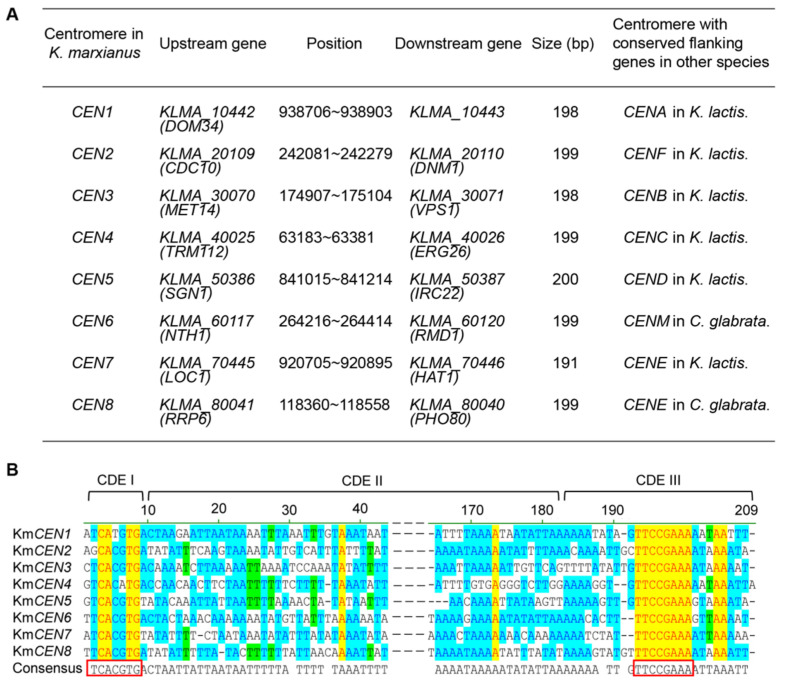
The positions and sequences of centromeres in *K. marxianus.* (**A**) The positions of centromeres in *K. marxianus*. The positions of centromeres refer to the positions in the genome of DMKU3-1042 [48]. (**B**) Alignments of centromeres in *K. marxianus.* The consensus sequences are in the red box. Color setup of the alignments: blue for conservative, green for similar, yellow for identical. The alignments were performed by Vector NTI (Thermo Fisher Scientific, Waltham, MA, USA).

A major aim of this study was to achieve the chromosomal rearrangements in *K. marxianus* by the Cre-loxP system. Centromeres are chromosomal regions that mediate kinetochore assembly and spindle attachment for faithful chromosome transmission during cell division [43]. Centromeres have profound effects on the efficiency and outcome of recombination, especially that involving large-scale chromosomal fragments [44]. However, positions of centromeres in *K. marxianus* have not been completely identified, which was an obstacle to the analysis of chromosomal rearrangements. Therefore, centromeres of *K. marxianus* were identified in the first place. Centromeric environments on either or both sides of centromeres are often conserved in *Candida glabrata*, *K. lactis* and *S. cerevisiae* [45]. Centromeres of chromosome V (Km*CEN5*) and VI (Km*CEN6*) of *K. marxianus* were characterized previously [46]. Genes adjacent to Km*CEN5* and Km*CEN6* were homologous to genes adjacent to chromosome D of *K. lactis* (Kl*CEND*) and chromosome M of *C. glabrata* (Cg*CENM*), respectively. Therefore, positions of centromeres in *K. marxianus* were predicted by conservation of flanking genes between *K. marxianus*, *C. glabrata* and *K. lactis* (Figure 1A). Same as the centromere in *K. lactis* [41] and *S. cerevisiae* [47], the centromere in *K. marxianus* was organized into three elements, the centromere DNA element I (CDEI), CDEII and CDEIII (Figure 1B). CDEI is 9 bp in length and contains a consensus sequence of TCACGTG (7 bp). CDEIII is 26 bp in length and contains a consensus sequence of TTCCGAAA. CDEII is AT-rich and separates CDEI and CEDIII. The length of CDEII ranges from 156 bp to 165 bp, which is twice the length of CDEII in *S. cerevisiae*. 

To verify the function of predicted centromeres in *K. marxianus*, the centromere was inserted into a highly unstable plasmid, LHZ881. LHZ881 contained an autonomously replicating sequence of *K. marxianus* (KmARS1) [49] and its mitotic stability was only 4.35% after seven-generation growth in the unselective medium (Table 1). Insertion of Km*CEN5* into LHZ881 increased the mitotic stability of the resultant plasmid (LHZ882) to 26.28% (Table 1). The stability of LHZ882 was substantially higher than that of LHZ881 but was still much lower than that of a CEN plasmid in *S. cerevisiae* or *K. lactis* [50,51]. In *K. lactis*, centromere and 50~100 bp flanking sequences were protected from the cleavage of DNase I or MNase, suggesting a role of flanking sequences in the centromeric function [52]. To investigate the influence of flanking sequences in *K. marxianus*, Km*CEN5* with 200 bp upstream sequence and 173 bp downstream sequence were inserted into LHZ881 to obtain LHZ887. The stability of LHZ887 was 82.82% after seven-generation growth in the unselective medium (Table 1), indicating flanking sequences were critical for the stable propagation of CEN plasmid in *K. marxianus*. Other centromeres, in company with their flanking sequences, were inserted into LHZ881, respectively. The mitotic stabilities of these plasmids ranged from 63.52% to 84.56%, corresponding to plasmid loss rates from 1.98% to 0.94% per generation (Table 1). These results indicated that predicted centromeres were functional in *K. marxianus*.

### 3.2. Establishment of a Cre-loxP System Controlled by a Leaky LAC4 Promoter and Unstable panARS Element in K. marxianus

**Figure 2:**
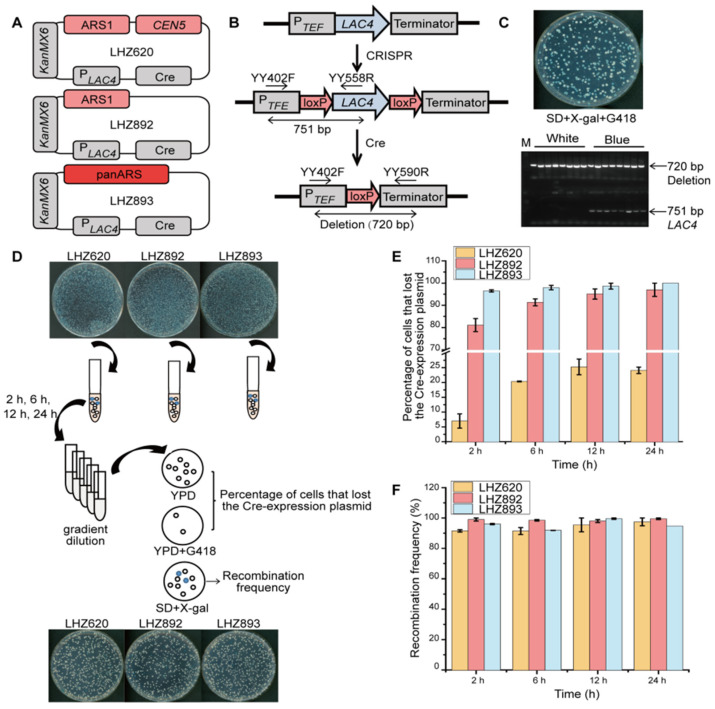
Establishment of a Cre-loxP system controlled by a *LAC4* promoter and panARS element in *K. marxianus*. (**A**) A schematic representation of three Cre-expressing plasmids. (**B**) A schematic representation of the deletion of the *LAC4* gene by the Cre-loxP system. (**C**) Identification of the deletion of the *LAC4* gene by colony PCR. A representative plate of LHP719 transformed with LHZ620 is shown above. White and blue colonies were subjected to colony PCR using YY402F/YY558R and YY402F/YY590R primer pairs. (**D**) The flow chart to measure the percentage of cells that lost the Cre plasmid and frequency of recombination after growing in unselective YPD medium. (**E**,**F**) The percentage of cells that lost the Cre plasmid (**E**) and the frequency of recombination at the *LAC4* locus (**F**) after growing in YPD for indicated times. The value represents mean ± standard errors (n = 3).

In our previous study, we showed that expression of Cre recombinase by a strong *INU1* promoter caused toxicity to *K. marxianus* cells and the toxicity was alleviated by using a leaky *LAC4* promoter in the glucose medium [18]. The *LAC4* promoter was induced by lactose or galactose and the activation could be repressed by glucose [18]. However, in contrast to the stringently repressed *GAL1* promoter in *S. cerevisiae* [53], the *LAC4* promoter was subjected to weak glucose repression, which led to a low-level leaky expression without inducers. Consistently, the β-galactosidase activity driven by the *LAC4* promoter in the glucose medium was 1/11 of that in the lactose medium and was 1/8 of that in the galactose medium in *K. marxianus*. Therefore, the alleviated toxicity of Cre to *K. marxianus* cells was attributed to the low-level expression of Cre driven by the leaky *LAC4* promoter [18]. In this study, the *LAC4* promoter was used to express Cre in a plasmid LHZ620, which also contained ARS1 and *CEN5* of *K. marxianus*, and a selective *KANMX6* marker (Figure 2A). All mediums contained glucose to reduce the expression of Cre.

To investigate whether the leaky expression of Cre was sufficient to induce recombination between loxP sites, LHZ620 was transformed into LHP719 in which the ORF of *LAC4* was flanked by two loxP sites (Figure 2B). Transformants were selected on the SD + X-gal + G418 plate, X-gal was hydrolyzed into blue precipitates by β-galactosidase, which was encoded by the *LAC4* gene [18]. As shown in Figure 2C, blue and white colonies were formed on the plate and subjected to colony PCR. As expected, the *LAC4* gene was deleted by Cre recombinase in white colonies (Figure 2C). In blue colonies, bands corresponding to the deletion of *LAC4* and the presence of intact *LAC4* were both detected. The results indicated that *LAC4* genes were gradually deleted by Cre recombinase during growth and that led to the mixture of blue and white cells in one colony. Since the prolonged presence of the Cre plasmid interfered with the genetic stability of host cells, LHP620 was optimized to facilitate the rapid loss of plasmids during growth in the unselective medium. To this end, *CEN5* and ARS1 in the LHP620 were replaced by a sole ARS1 of *K. marxianus* to obtain LHZ892, or by a panARS element to obtain LHZ893 (Figure 2A). A panARS element is a short ARS sequence identified in *K. lactis*. PanARS allows episomal plasmid maintenance in diverse species of budding yeast. However, loss frequencies per generation of panARS-based plasmids ranged from 5% to 20% [35], suggesting panARS-based plasmids would not be as stable as centromere-based plasmids during propagation in *K. marxianus* (Table 1). LHZ620, LHZ892 and LHZ893 were transformed into LHP719 separately. Colonies formed on SD + X-gal + G418 plates were mixed and grown in the unselective YPD medium for indicated times. Cells were then spread onto YPD and YPD + G418 plates to calculate the percentage of cells that lost the Cre-expressing plasmid or spread onto SD + X-gal plates to calculate the recombinant frequency (Figure 2D). After growing in YPD for 24 h, the percentage of cells that lost LHZ620 was 24%, and the percentage of cells that lost LHZ892 and LHZ893 were 97% and 100%, respectively. Notably, the percentage of cells that lost LHZ893 reached 96% after only 2 h growth in YPD (Figure 2E). Therefore, panARS-based LHZ893 was an unstable plasmid during propagation and that facilitated the rapid clearance of Cre-plasmid from the host. The recombination frequency was calculated by the percentage of white colonies. After growing in YPD, the highest recombination frequency of cells transformed with LHZ620 was 97%, and those of cells transformed with LHZ892 and LHZ893 were 99% (Figure 2F). LHZ893 promoted efficient recombination between loxP sites, and it could be quickly removed from the cells by growing in the unselective medium. Therefore, LHZ893 containing Cre recombinase behind a leaky *LAC4* promoter and an unstable panARS element was recognized as a novel and effective Cre-expressing plasmid in *K. marxianus* and was applied in the following studies. 

### 3.3. Deletion, Inversion and Interchromosomal Rearrangements Mediated by loxPsym and Cre in K. marxianus

**Figure 3:**
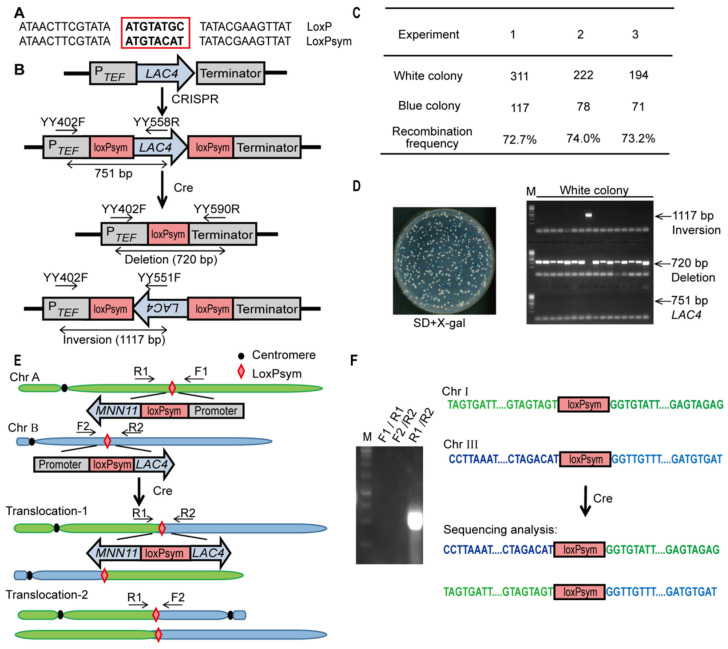
Recombination between loxPsym sites. (**A**) Sequences of loxP and loxPsym sites. The 8-bp central spacer regions are shown in the red box. (**B**) A schematic representation of recombination between loxPsym sites flanking *LAC4* ORF. (**C**) The recombination efficiency between loxPsym sites. The efficiency was indicated by the percentage of white colonies on SD + X-gal plates. (**D**) Identification of type of recombination by PCR. Intact *LAC4* locus produced a band of 751 bp. Inversion and deletion of *LAC4* ORF produced bands of 1117 bp and 720 bp, respectively. (**E**) A schematic representation of interchromosomal rearrangements mediated by the recombination between loxPsym sites. (**F**) Identification of rearrangements by PCR. A band produced by primer R1 and R2 indicated interchromosomal rearrangements. Fused sequences from Chr I and III through loxPsym sites are shown on the right.

loxPsym is a palindromic loxP site with an 8-bp symmetric spacer region (Figure 3A) [54]. loxPsym sites lack the directionality of canonical loxP sites, and thus Cre-mediated recombination between two loxPsym sites leads to either deletion or inversion of internal sequence (Figure 3B). The recombination efficiency between loxPsym sites at the *LAC4* locus was investigated by using the panARS-based plasmid LHZ893. Transformants were grown in YPD medium for 12 h and then spread onto the SD + X-gal plate. The average recombination efficiency was 73% (Figure 3C). Sixteen white colonies were subjected to colony PCR to verify the type of recombination. One colony contained the inversion of the *LAC4* gene, and the rest of the colonies contained the deletion of *LAC4* (Figure 3D). The deletion can occur after the inversion at the same locus, but inversion could not occur after deletion since only one loxPsym site is preserved. Therefore, the inversed loci might be gradually turned into deleted loci in the presence of Cre, which led to the high frequency of deletion [54]. 

Next, the large-scale interchromosomal rearrangements between loxPsym sites were investigated. In LHP1043, one loxPsym site was inserted in front of the *LAC4* ORF in Chr III and the other site was placed in front of the *MNN11* ORF in Chr I (Figure 3E). LHZ893 was transformed into LHP1043, and cells were spread onto SD + X-gal after growing in unselective medium. Recombination between these loxPsym sites resulted in the rearrangement between Chr I and III. In one type of rearrangement, ORFs of *LAC4* and *MNN11* were put together in the opposite direction while promoters of both genes were fused on the other chromosome. This rearrangement impaired the expression of *LAC4* and led to the formation of white colonies on the X-gal plate. In the other type of rearrangement, promoters of *LAC4* and *MNN11* were swapped. The rearrangement produced one dicentric chromosome and an acentric chromosome, which led to genome instability and growth defects (Figure 3E) [55,56]. White colonies were picked and subjected to colony PCR. A band produced by primers located on different chromosomes suggested the interchromosomal rearrangement. Additionally, the fused sequence from Chr I and III was verified by sequencing (Figure 3F). Results suggested Cre recombinase could mediate deletion, inversion and interchromosomal rearrangements through loxPsym sites in *K. marxianus*.

### 3.4. Chromosomal Rearrangement between 16 loxPsym Sites by Cre in K. marxianus

**Figure 4:**
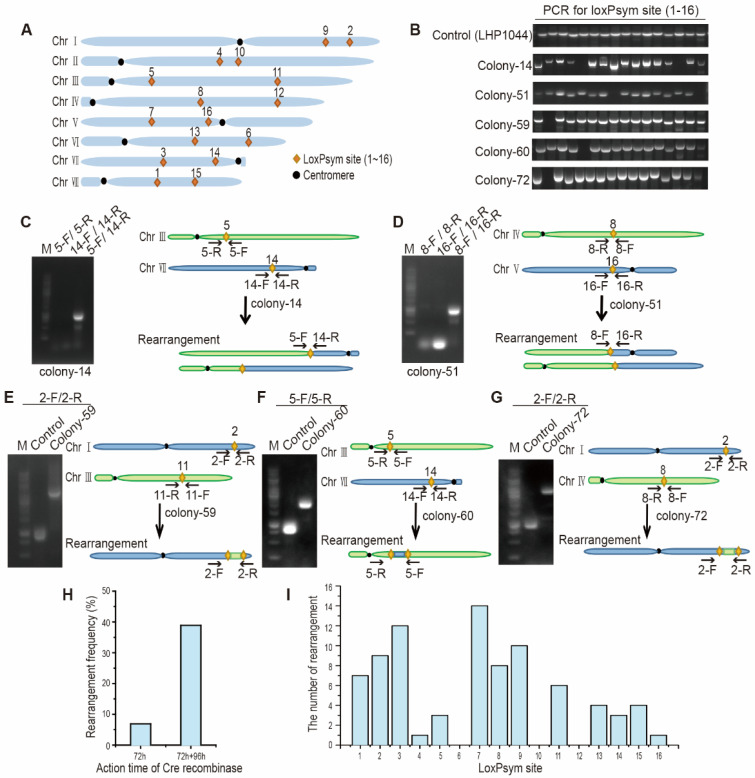
Rearrangements between multiple loxPsym sites. (**A**) Distribution of 16 loxPsym sites among eight chromosomes. (**B**) Identification of rearrangements in different colonies by PCR using 16 pairs of primers. LHP1044 served as a control. Five colonies displaying missing bands at certain site(s) are shown below. (**C**–**G**). Verification of interchromosomal rearrangement in five colonies by PCR. PCR products produced by the primer pair 5-F/14-R, suggested the translocation in colony 14 (**C**). PCR product produced by the primer pair 8-F/16-R, suggested the translocation in colony 51 (**D**). PCR product produced by 2-F/2-R in colony 59 (**E**) or colony 72 (**G**) was bigger than that in LHP1044 control, and PCR product produced by 5-F/5-R in colony 60 was bigger than that in the control (**F**), suggesting the rearrangements at these sites. (**H**) Rearrangement frequency between loxPsym sites with an extended action time of Cre. In all, 72 h and 72 h + 96 h indicated the time of growth on YPD + G418 plates. (**I**) The number of rearrangement events identified at different loxPsym sites.

To evaluate the efficiency of rearrangements between multiple loxPsym sites, LHP1044 containing 16 loxPsym sites distributed among eight chromosomes was constructed (Figure 4A). LHP1044 was transformed with LHZ893 and transformants were grown on the YPD + G418 plate for 72 h. Transformants were then grown in YPD for 12 h and spread onto YPD plates. Seventy-two colonies were randomly selected and subjected to PCR using 16 pairs of primers corresponding to 16 loxPsym sites. As a control, PCR of LHP1044 produced 16 bands for 16 sites. Among 72 colonies, 5 colonies failed to obtain the expected band at one or more sites, suggesting rearrangement occurred at these sites (Figure 4B). Colony 14 contained a translocation between Chr III (site 5) and Chr VII (site 14), as a PCR product was obtained by using primers of site 5 and 14 (Figure 4C). Colony 51 contained a translocation between Chr IV (site 8) and Chr V (site 16), as a PCR product was obtained by using primers of site 8 and 16 (Figure 4D). Both translocations occurred through loxPsym sites. In the other three colonies, primer pairs at certain sites produced bands bigger than expected, suggesting that an insertion occurred in the sites (Figure 4E–G). In colony 59, a fragment adjacent to loxPsym site 11 in Chr III was inserted into the position at loxPsym site 2 in Chr I. In colony 60, a fragment adjacent to loxPsym site 14 in Chr VII was inserted into the position at loxPsym site 5 in Chr III. In colony 72, a fragment adjacent to loxPsym site 8 in Chr IV was inserted into the position at loxPsym site 2 in Chr I (Figure 4E–G). The results indicated that multiple loxPsym across the genome could mediate large-scale translocations and cause insertions of small fragments around the site. However, the recombination frequency was only 6.9% (5 colonies/72 colonies). To improve the recombination frequency, the action time of Cre recombinase was extended by growing on the selective YPD + G418 plate for an extra 96 h and the frequency increased to 38.9% (28 colonies/72 colonies) (Figure 4H). Among a total of 144 colonies identified, 33 colonies exhibited rearrangements at certain sites. In these colonies, no rearrangement was detected at site 6, 10 and 12, suggesting these sites were not prone to loxPsym-mediated recombination (Figure 4I). Meanwhile, 14, 12 and 10 events of rearrangement occurred at site 7, 3 and 9, respectively, suggesting these sites were hotspots for recombination (Figure 4I). The high-order structure of the chromatin around loxPsym sites or surrounding genes might determine the bias for the recombination [57].

## 4. Discussion

In this study, centromeres of eight chromosomes in *K. marxinaus* were identified. Around 200 bp flanking sequences of Km*CEN5* were indispensable for maintaining mitotic stability (Table 1). In *K. lactis*, a 100 bp AT-rich (82%~95%) element, named K1CDE0, was found ±150 bp upstream of KlCDEI and was important for complete centromere function [41,52]. The high AT content of KlCDE0 was predicted to have an anti-bend structure, which allows the binding of specific centromere proteins [41]. In *K. marxianus*, AT-rich regions (89%~100%) were also found upstream of Km*CEN1*, Km*CEN2*, Km*CEN3*, Km*CEN7* and Km*CEN8*. The regions were located 24~225 bp upstream of CDEI elements. Notably, the regions were mainly composed of A residues, as regions upstream of Km*CEN1*, Km*CEN2*, Km*CEN3*, Km*CEN7* and Km*CEN8* contained 67%, 79%, 79%, 71% and 66% of A residue, respectively. However, no AT-rich regions (>80%) were identified within 400 bp upstream of Km*CEN2*, Km*CEN5* and Km*CEN6*. Meanwhile, bias toward the A residue was not found in the upstream sequence of these centromeres. Therefore, the CDE0 element was not well-conserved in *K. marxianus*, suggesting a difference in maintaining centromere function between *K. lactis* and *K. marxianus*. 

In yeast, some episomal plasmids driven by induced promoters were employed to control the transient expression of the Cre recombinase. In *Candida albicans*, a *MET3* promoter induced by methionine was used to control the expression of Cre [58]. In *Hansenula polymorpha*, Cre recombinase was used to generate a recyclable marker system under the regulation of a methanol-inducible FMD promoter [59]. However, stringent inducible promoters are not available in some yeasts. Therefore, unstable vectors were applied to regulate the expression of Cre recombinase in some cases. In *Xanthophyllomyces dendrorhous*, transient expression of the Cre recombinase was controlled by a genetically unstable vector that did not include a yeast replicative origin [28]. In *Aureobasidium melanogenum*, the Cre-expressing plasmid contained an exogenous ARS element from *Ustilago maydis* to keep its temporary stability [29]. In our study, to reduce the mitotic stability of the Cre-expressing plasmid, panARS from *K. lactis* was included in the plasmid. PanARS was capable of supporting efficient transformation on the selective medium (Figure 2D). Meanwhile, the percentage of cells that lost the Cre-expressing plasmid reached 96.5% after 2 h growth in the unselective medium, indicating panARS1 facilitated the rapid removal of the Cre-expressing plasmid from the host cell (Figure 2E). 

To reduce the toxicity of Cre to the *K. marxianus*, a low level of Cre was expected to be produced by the *LAC4* promoter in the presence of glucose. As shown by the high percentage of white colonies formed on the YPD plate (2% glucose), 99% efficiency of recombination at the *LAC4* loci was achieved by the leakage of Cre recombinase. The result suggested that low-level Cre was sufficient to promote recombination while reducing the toxicity to host cells. Similar cases were reported in other species. Cre was toxic to *S. pombe* cells [17] and Cre expressed by a low-strength nmt81 promoter was capable of producing efficient recombination [60]. Low levels of Cre activity permitted recombination without concomitant toxicity in mammalian cells [16]. Therefore, the level of Cre recombinase needs to be appropriately monitored to balance the efficiency and toxicity.

In this study, chromosomal rearrangements were identified by a PCR-based assay. The PCR assay was repeated by using the genomic DNA extracted from different candidate colonies to confirm the conclusion. However, the PCR assay could not identify rearrangements beyond the scope of primers, and it also had trouble identifying small insertion-deletion. In further experiments, PFGE paired with Southern blot analysis, high-throughput analysis methods including CGHarray, SNP microarray and second-generation sequencing might be applied to achieve an accurate map of chromosomal rearrangements in the strains.

Chromosomal rearrangements, including deletions, duplications, inversions and translocations, are important for human health because they are involved in the pathogenesis of many diseases. Approximately 15–20% of patients with intellectual disability and autism spectrum disorders have a clinically relevant chromosome rearrangement [61]. Recent studies reported that the deletion and the duplication in genomes are potentially pathogenic factors in dystonia and parkinsonism [62]. In addition to driving many diseases, chromosomal rearrangements also contribute to genetic diversity and evolution, which are the basics of phenotypic diversification and environmental adaptability. For example, the translocation between Chr XV and ChrXVI involving the promoter of *ADH1* and the gene *SSU1* improved the adaptation to sulfite of *S. cerevisiae* [63]. Multiple gene-editing technologies have been developed to decipher the mechanism, genetic dependencies, hotspots and phenotypic effects of chromosomal rearrangements.

The Cre-loxP system developed in this study could not only mediate the deletion and inversion of a single gene but also promote large-scale chromosome translocation. Compared to highly efficient recombination around a single gene, the efficiencies of translocations were low, as the recombination efficiency dropped with increasing distance between recombination sites [64]. The efficiencies of rearrangement were different at various loxPsym sites, which might be related to the accessibility of surrounding chromatin structure and effects of genes in the context. Large-scale rearrangement breaks the long-standing chromosomal homeostasis and presents a unique transcription pattern that affects the physiological and biochemical indexes of cells [65,66,67]. Therefore, *K. marxianus* carrying multiple loxPsym sites among eight chromosomes might be subjected to iterative cycles of large-scale intrachromosomal and interchromosomal recombination. A study of resultant mutants will provide valuable information on the chromatin function. Meanwhile, mutants will compose useful libraries for screens of desirable phenotypes, including resistance to stresses, and productions of heterologous proteins, ethanol and chemicals.

## Figures and Tables

**Table 1:** The mitotic stability of plasmid carrying centromere of *K. marxianus*.

Plasmid	Centromere	Flanking Sequences	N_0_ (%)	N_7_ (%)	Loss per Generation (%)
LHZ881	-	−	18.57	4.35	-
LHZ882	*CEN5*	−	64.48	26.28	12.5
LHZ883	*CEN1*	+	90.87	83.05	1.40
LHZ884	*CEN2*	+	89.52	78.01	1.98
LHZ885	*CEN3*	+	95.79	84.56	1.87
LHZ886	*CEN4*	+	70.98	63.52	0.94
LHZ887	*CEN5*	+	91.23	82.82	1.32
LHZ888	*CEN6*	+	92.52	81.90	1.75
LHZ889	*CEN7*	+	84.10	77.50	1.16
LHZ890	*CEN9*	+	82.19	74.34	1.45

N_0_ and N_7_ represent the percentages of cells containing the plasmid after 0 and 7 generations of unselective growth, respectively. The value represents the average from three biological repeats.

## Data Availability

The data presented in this study are available upon request from the corresponding author.

## Supplementary Materials

The following supporting information can be downloaded at: https://www.mdpi.com/article/10.3390/microorganisms10061240/s1, Supplementary File S1: Table S1. Strains used in this study; Supplementary File S2: Table S2. Plasmids used in this study; Supplementary File S3: Table S3: Primers used in this study; Supplementary File S4: Sequences of LHZ881; Supplementary File S5: Sequences of LHZ882.
