# Supplementary material for: Establishment of a Cre-loxP System Based on a Leaky LAC4 Promoter and an Unstable panARS Element in Kluyveromyces marxianus
Source: Microorganisms. 2022 Jun 17;10(6):1240. doi: 10.3390/microorganisms10061240 (PMC9227491; doi:10.3390/microorganisms10061240)
Supplement: Supplementary file 1 [file microorganisms-10-01240-s001.zip › Supplementary File S1.pdf]

Table S1. Strains used in this study

Name	Genotype	Sources
FIM-1	wild type	[31]
FIM-1ΔU	*ura3* Δ	[31]
αFIM-1ΔU	*MATα*, *ura3* Δ	[36]
αFIM-1ΔUΔH	*MATα*, *ura3* Δ, *his3* Δ	[36]
LHP719	*MATα*, *ura3* Δ, *his3* Δ, *lac4* Δ::P_{TEF}-loxP-*LAC4* -loxP	This study
LHP756	*MATα*, *ura3* Δ, *his3* Δ, *lac4* Δ::P_{TEF}-loxPsym-*LAC4* -loxPsym	This study
LHP758	*MATα*, *ura3* Δ, *his3* Δ, *lac4* Δ::P_{TEF}-loxPsym-*LAC4*	This study
LHP1043	*MATα*, *ura3* Δ, *his3* Δ, *lac4* Δ*::P_{TEF}-loxPsym-LAC4* ,	This study
LHP1044	*MATα*, *ura3* Δ, loxpsym(16)*	This study

* position of 16 loxPsym: Chr I (1451427, 1673871), Chr II (717538, 896924), Chr III (1219332, 326046), Chr IV (1172245, 704908), Chr V (346042, 756338), Chr VI (1069906, 753935), Chr VII (409354, 689479), Chr VIII (610289, 440003)
