# Supplementary material for: Establishment of a Cre-loxP System Based on a Leaky LAC4 Promoter and an Unstable panARS Element in Kluyveromyces marxianus
Source: Microorganisms. 2022 Jun 17;10(6):1240. doi: 10.3390/microorganisms10061240 (PMC9227491; doi:10.3390/microorganisms10061240)
Supplement: Supplementary file 1 [file microorganisms-10-01240-s001.zip › Supplementary File S2.pdf]

Table S2. Plasmids used in this study

| Name | Description | Backbone | application | Sources |
|---|---|---|---|---|
| LHZ881 | KmARS1, *HphMX4* | / | Vector backbone for centromere plasmid | [38] |
| LHZ882 | KmARS1, Km*CEN5* , *HphMX4* | LHZ881 | Centromere plasmid for verification the function of KmCEN5 | |
| LHZ883 | KmARS1, Km*CEN1* and flanking sequence, *HphMX4* | LHZ881 | Centromere plasmid for verification the function of KmCEN1 and its flanking sequence | |
| LHZ884 | KmARS1, Km*CEN2* and flanking sequence, *HphMX4* | LHZ881 | Centromere plasmid for verification the function of KmCEN2 and its flanking sequence | |
| LHZ885 | KmARS1, Km*CEN3* and flanking sequence, *HphMX4* | LHZ881 | Centromere plasmid for verification the function of KmCEN3 and its flanking sequence | |
| LHZ886 | KmARS1, Km*CEN4* and flanking sequence, *HphMX4* | LHZ881 | Centromere plasmid for verification the function of KmCEN4 and its flanking sequence | |
| LHZ887 | KmARS1, Km*CEN5* and flanking sequence, *HphMX4* | LHZ881 | Centromere plasmid for verification the function of KmCEN5 and its flanking sequence | |
| LHZ888 | KmARS1, Km*CEN6* and flanking sequence, *HphMX4* | LHZ881 | Centromere plasmid for verification the function of KmCEN6 and its flanking sequence | |
| LHZ889 | KmARS1, Km*CEN7* and flanking sequence, *HphMX4* | LHZ881 | Centromere plasmid for verification the function of KmCEN7 and its flanking sequence | |
| LHZ890 | KmARS1, Km*CEN8* and flanking sequence, *HphMX4* | LHZ881 | Centromere plasmid for verification the function of KmCEN8 and its flanking sequence | |
| LHZ620 | KmARS1, Km*CEN5* , PLAC4-Cre, *KanMX6* | / | / | [34] |
| LHZ892 | KmARS1, Cre, *KanMX6* | LHZ620 | Cre plasmid mediated by ARS1 | |
| LHZ893 | panARS, Cre, *KanMX6* | LHZ620 | Cre plasmid mediated by panARS | |
| LHZ531 | ARS1, Cas9, gRNA, *URA3* | / | CRIPSR vector | [37] |
| LHZ891 | gRNA-RHY101F/RHY101R | LHZ531 | CRISPR plasmid for the deletion of *LAC4* and its promoter | |
| LHZ894 | *LAC4* upstream sequence-*TEF* promoter -loxP-*LAC4* -loxP sequence-*LAC4* downstream sequence | PMD18-T | Providing donor sequence to replace *LAC4* and its promoter with a cassette of TEF promoter -loxP-*LAC4* -loxP | |
| LHZ895 | *LAC4* upstream sequence-*TEF* promoter-loxPsym-*LAC4* -loxPsym sequence-*LAC4* downstream sequence | PMD18-T | Providing donor sequence to replace *LAC4* and its promoter with a cassette of TEF promoter and loxPsym-*LAC4* -loxPsym | |
| LHZ896 | *LAC4* upstream sequence-*TEF* promoter-loxPsym-*LAC4* -*LAC4* downstream sequence | PMD18-T | Providing donor sequence to replace *LAC4* and its promoter with a cassette of TEF promoter and loxPsym-*LAC4* | |
| LHZ897 | gRNA-RHY102F/RHY102R | LHZ531 | CRISPR plasmid for insertion of loxPsym site at Chr I (747278bp) | |
| LHZ898 | gRNA-RHY103F/RHY103R | LHZ531 | CRISPR plasmid for the insertion of loxPsym site at Chr VIII (440003) | |
| LHZ899 | gRNA-RHY104F/RHY104R | LHZ531 | CRISPR plasmid for the insertion of loxPsym site at Chr VII (409354) | |
| LHZ900 | gRNA-RHY105F/RHY105R | LHZ531 | CRISPR plasmid for the insertion of loxPsym site at Chr I (1673871) | |
| LHZ901 | gRNA-RHY106F/RHY106R | LHZ531 | CRISPR plasmid for the insertion of loxPsym site at Chr II (896924) | |
| LHZ902 | gRNA-RHY107F/RHY107R | LHZ531 | CRISPR plasmid for the insertion of loxPsym site at Chr I (1451427) | |
| LHZ903 | gRNA-RHY108F/RHY108R | LHZ531 | CRISPR plasmid for the insertion of loxPsym site at Chr III (326046) | |
| LHZ904 | gRNA-RHY109F/RHY109R | LHZ531 | CRISPR plasmid for the insertion of loxPsym site at Chr IV (704908) | |
| LHZ905 | gRNA-RHY110F/RHY110R | LHZ531 | CRISPR plasmid for the insertion of loxPsym site at Chr V (346042) | |
| LHZ906 | gRNA-RHY111F/RHY111R | LHZ531 | CRISPR plasmid for the insertion of loxPsym site at Chr VI (1069906) | |
| LHZ907 | gRNA-RHY112F/RHY112R | LHZ531 | CRISPR plasmid for the insertion of loxPsym site at Chr III (1219332) | |
| LHZ908 | gRNA-RHY113F/RHY113R | LHZ531 | CRISPR plasmid for the insertion of loxPsym site at Chr IV (1172245) | |
| LHZ909 | gRNA-RHY114F/RHY114R | LHZ531 | CRISPR plasmid for the insertion of loxPsym site at Chr V (756338) | |
| LHZ910 | gRNA-RHY115F/RHY115R | LHZ531 | CRISPR plasmid for the insertion of loxPsym site at Chr VI (753935) | |
| LHZ911 | gRNA-RHY116F/RHY116R | LHZ531 | CRISPR plasmid for the insertion of loxPsym site at Chr VII (689479) | |
| LHZ912 | gRNA-RHY117F/RHY117R | LHZ531 | CRISPR plasmid for the insertion of loxPsym site at Chr II (717538) | |
| LHZ913 | gRNA-RHY118F/RHY118R | LHZ531 | CRISPR plasmid for the insertion of loxPsym site at Chr VIII (610289) | |
