# Supplementary material for: Establishment of a Cre-loxP System Based on a Leaky LAC4 Promoter and an Unstable panARS Element in Kluyveromyces marxianus
Source: Microorganisms. 2022 Jun 17;10(6):1240. doi: 10.3390/microorganisms10061240 (PMC9227491; doi:10.3390/microorganisms10061240)
Supplement: Supplementary file 1 [file microorganisms-10-01240-s001.zip › Supplementary File S3.pdf]

Table S3. Primers used in this study

Name	Sequence(5'-3')	Description	Alias
HphMX4-F	CATCACATCCGAACATAAACAACCATGGGTAAA AAGCCTGAACTCACCGCGACG	Forward primer PCR for amplifying HphMX4 from pCloneHyg1, and then replace the KANMX6 of pFA6a-13Myc-KANMX6 by HphMX4 to obtain pFA6a-13Myc-HphMX4	
HphMX4-R	CTTTTTATTGTCAGTACTGATTATTCCTTTGCCC TCGGACGAGTGCTGGGG	Reverse primer PCR for amplifying HphMX4 from pCloneHyg1, and then replace the KANMX6 of pFA6a-13Myc-KANMX6 by HphMX4 to obtain pFA6a-13Myc-HphMX4	
KmARS1-F	GAACGCGGCCGCCAGCTGAAGCTTATCGATTGA AGTTTTGTCCAACTATCCACTATG	Forward primer PCR for amplifying KmARS1 from genomic DNA of FIM-1, and ligating with ADH1 by overlap extension PCR, then inserting into Sma I and Hind III site of pFA6a-13Myc-HphMX4 to obtain LHZ881	
KmARS1-R	CTATTTCATTTACCATCATTTCTTCGTCGACCCG CGGTTTTAACGATATATCATTTTCAA	Reverse primer PCR for amplifying KmARS1 from genomic DNA of FIM-1, and ligating with ADH1 by overlap extension PCR, then inserting into Sma I and Hind III site of pFA6a-13Myc-HphMX4 to obtain LHZ881	
ADH1-F	TTGAAAATGATATATCGTTAAAACCGCGGGTCG ACGAAGAAATGATGGTAAATGAAATAG	Forward primer PCR for amplifying ADH1 from genomic DNA of S288C, and ligating with KmARS1 by overlap extension PCR, then inserting into Sma I and Hind III site of pFA6a-13Myc-KANMX6 to obtain LHZ881	
ADH1-R	GCCTCGAGTACCGGTACCGCTACCCGGGAGTTG ATTGTATGCTTGGTATAGCTTG	Reverse primer PCR for amplifying ADH1 from genomic DNA of S288C, and ligating with KmARS1 by overlap extension PCR, then inserting into Sma I and Hind III site of pFA6a-13Myc-KANMX6 to obtain LHZ881	
cen5-F	TAAATTGAAAATGATATATCGTTAAAACCGCGG GTCACGTGTATACAAATTATTAATTTTAAAAC	Forward primer PCR for amplifying KmCEN5 from genomic DNA of FIM-1, and then inserting into Sal I site of LHZ881 vector to obtain LHZ882	
cen5-R	CATTTACCATCATTTCTTCGTCGACGGATCCTAT TTTACTTTCGGAACAACTTTTTAAC	Reverse primer PCR for amplifying KmCEN5 from genomic DNA of FIM-1, and then inserting into Sal I site of LHZ881 vector to obtain LHZ882	
FS-cen1-F	AAAATGATATATCGTTAAAAATAATAAAATATA ATTTTTACATAGTATAACTCTAAAC	Forward primer PCR for amplifying KmCEN1 and flanking sequence from genomic DNA of FIM-1, and then inserting into Sal I site of LHZ881 vector	
FS-cen1-R	CATTTACCATCATTTCTTCGTCGACTTTAATGAT TAATTGTTACATTTGGTATAAG	Reverse primer PCR for amplifying KmCEN1 and flanking sequence from genomic DNA of FIM-1, and then inserting into Sal I site of LHZ881 vector	
FS-cen2-F	TGAAAATGATATATCGTTAAAATAAATAGAAAT AAAGTTTTTATATAAAAGAATAAAAG	Forward primer PCR for amplifying KmCEN2 and flanking sequence from genomic DNA of FIM-1, and then inserting into Sal I site of LHZ881 vector	
FS-cen2-R	ATTTCATTTACCATCATTTCTTCCTTAATAACTC TTATAAACTGATATAATTTTGTTTA	Reverse primer PCR for amplifying KmCEN2 and flanking sequence from genomic DNA of FIM-1, and then inserting into Sal I site of LHZ881 vector	
FS-cen3-F	AATTGAAAATGATATATCGTTAAAATTCGTTAC ATAAACAACACGCTTTTATAAAC	Forward primer PCR for amplifying KmCEN3 and flanking sequence from genomic DNA of FIM-1, and then inserting into Sal I site of LHZ881 vector	
FS-cen3-R	ATTTACCATCATTTCTTCGTCGACAATAAAACAA AATAAAATAAAAATAAAATAAAAAT	Reverse primer PCR for amplifying KmCEN3 and flanking sequence from genomic DNA of FIM-1, and then inserting into Sal I site of LHZ881 vector	
FS-cen4-F	AATTGAAAATGATATATCGTTAAAAATCGTTAT TCTTTATTCTTTGCAATTAAC	Forward primer PCR for amplifying KmCEN4 and flanking sequence from genomic DNA of FIM-1, and then inserting into Sal I site of LHZ881 vector	
FS-cen4-R	ATTTCATTTACCATCATTTCTTCAAAAAATAAAT AAAATAAAATAAAATAAAATAAAAT	Reverse primer PCR for amplifying KmCEN4 and flanking sequence from genomic DNA of FIM-1, and then inserting into Sal I site of LHZ881 vector	
FS-cen5-F	TTGAAAATGATATATCGTTAAAAGTCAACCGCA ACACCACCTATTTCTAAGAGGAGAG	Forward primer PCR for amplifying KmCEN5 and flanking sequence from genomic DNA of FIM-1, and then inserting into Sal I site of LHZ881 vector	
FS-cen5-R	TATTTCATTTACCATCATTTCTTCGTCGACGAGC TCCTTTCATTTCTGATAAAAGTAAGG	Reverse primer PCR for amplifying KmCEN5 and flanking sequence from genomic DNA of FIM-1, and then inserting into Sal I site of LHZ881 vector	
FS-cen6-F	AATTGAAAATGATATATCGTTAAAATAGTGTGT GGAACTTATAATTAAATACTTG	Forward primer PCR for amplifying KmCEN6 and flanking sequence from genomic DNA of FIM-1, and then inserting into Sal I site of LHZ881 vector	
FS-cen6-R	CATTTACCATCATTTCTTCGTCGACATCTAAATA AATATAGGATGCAAAAGC	Reverse primer PCR for amplifying KmCEN6 and flanking sequence from genomic DNA of FIM-1, and then inserting into Sal I site of LHZ881 vector	
FS-CEN7-F	AATTGAAAATGATATATCGTTAAAACTCTACCC TTTTCATTCCAAAAAGT	Forward primer PCR for amplifying KmCEN7 and flanking sequence from genomic DNA of FIM-1, and then inserting into Sal I site of LHZ881 vector	
FS-CEN7-R	CTATTTCATTTACCATCATTTCTTCAATTAAGTT AAAACGCCATAAAATATG	Reverse primer PCR for amplifying KmCEN7 and flanking sequence from genomic DNA of FIM-1, and then inserting into Sal I site of LHZ881 vector	
FS-cen8-F	ATTGAAAATGATATATCGTTAAAAAAATATTAA AGCATTGAAATAATAATAAATACAAT	Forward primer PCR for amplifying KmCEN8 and flanking sequence from genomic DNA of FIM-1, and then inserting into Sal I site of LHZ881 vector	
FS-cen8-R	CTATTTCATTTACCATCATTTCTTCAATTTTCCA AAAAAAATGTCATAAATCG	Reverse primer PCR for amplifying KmCEN8 and flanking sequence from genomic DNA of FIM-1, and then inserting into Sal I site of LHZ881 vector	
ARS1-F	GAACGCGGCCGCCAGCTGAAGCTTATCGATTGA AGTTTTGTCCAACTATC	Forward primer PCR for amplifying KmARS1from LHZ881, and then inserting into Pvu II and Sal I sites of LHP620	
ARS1-R	CTACCTGGCCTGGATGGCTGTCGACGGATCCTT TTAACGATATATCATTTTC	Reverse primer PCR for amplifying KmARS1from LHZ881, and then inserting into Pvu II and Sal I sites of LHP620	
panARS-F	GAACGCGGCCGCCAGCTGAAGCTTTCAACATCT TTGGATAATATCAGAATGAG	Forward primer PCR for amplifying panARS from the genomic DNA of ATCC8585, and then inserting into Pvu II and Sal I sites of LHP620	
panARS-R	CTACCTGGCCTGGATGGCTGTCGACTAGTGCTG ATTATGATTTGACGTTTATATAC	Reverse primer PCR for amplifying panARS from the genomic DNA of ATCC8585, and then inserting into Pvu II and Sal I sites of LHP620	
YY402F	TTGTTGCTACACCGTTCACTACTTG	PCR for identifying the LAC4 gene	
YY551F	TTGCTAACAAAATAAACTTGAACACG	PCR for identifying the inversion of LAC4 gene	
YY590R	CATTATCGCATAAGACAATCGGCAC	PCR for identifying the deletion of LAC4 gene	
YY558R	TCAAGAGGTGCGTCAAACAACGC	PCR for identifying the LAC4 gene	
RHY101F	TCA CTACTTGAAGTCGAGTGTGA	gRNA for LHZ891 CRISPR plasmid	
RHY101R	AAC TCACACTCGACTTCAAGTAG	gRNA for LHZ891 CRISPR plasmid	
RHY102F	TCA GATAGAATAT TTGGTAGTAG	gRNA for LHZ897 CRISPR plasmid	
RHY102R	AAC CTACTACCAA ATATTCTATC	gRNA for LHZ897 CRISPR plasmid	
RHY102-up-F	GAATGAAACACACAAATCTCCCTCC	PCR for amplifying the upstream sequence of Chr I (747278bp)	F1
RHY102-up-R	GAAAGTGCTCTACTCTGCTCTTAATACACCATA ACTTCGTATAATGTACATTATACGAAGTTATACT ACTACCAAATATTCTATCAAATCACTAAGG	PCR for amplifying the upstream sequence of Chr I (747278bp)	
RHY102-down-F	GGTGTATTAAGAGCAGAGTAGAGCACTTTC	PCR for amplifying the downstream sequence of Chr I (747278bp)	
RHY102-down-R	CTGACAACTCCTTCCTTTCAGCCTC	PCR for amplifying the downstream sequence of Chr I (747278bp)	R1
RHY103F	TCACATTGCTATGGAATGCAGCT	gRNA for LHZ898 CRISPR plasmid	
RHY103R	AACAGCTGCATTCCATAGCAATG	gRNA for LHZ898 CRISPR plasmid	
RHY103-up-F	ATGCTATGGTCAAGAAGAGAAAAGC	PCR for amplifying the upstream sequence of Chr VIII (440003)	15-F

RHY103-up-R	TCATTGCTATGGAATGCAGCTTGATAACTTCGTATAATGTACATTATACGAAGTTATGTGTTTATGTAATATTTCTAAATATGTAATAAAAG	PCR for amplifying the upstream sequence of Chr VIII (440003)	
RHY103-down-F	CAAGCTGCATTCCATAGCAATGA	PCR for amplifying the downstream sequence of Chr VIII (440003)	
RHY103-down-R	TACCTCCAACTCCTGAAAAGGACAC	PCR for amplifying the downstream sequence of Chr VIII (440003)	15-R
RHY104F	TCAGAATTGAATTTAACGAACCT	gRNA for LHZ899 CRISPR plasmid	
RHY104R	AACAGGTTCGTTAAATTCAATTC	gRNA for LHZ899 CRISPR plasmid	
RHY104-up-F	CAACCACAATCTACCGTTTCATCTG	PCR for amplifying the upstream sequence of Chr VII (409354)	3-F
RHY104-up-R	ATAAAAATGTCATAAATGGCTCTCCATAACTTCGTATAATGTACATTATACGAAGTTATGAGGTTCGTTAAATTCAATTCGATTTAAC	PCR for amplifying the upstream sequence of Chr VII (409354)	
RHY104-down-F	GGAGAGCCATTTATGACATTTTTAT	PCR for amplifying the downstream sequence of Chr VII (409354)	
RHY104-down-R	TTTCCGTTCTATTTTCGTTTATTTC	PCR for amplifying the downstream sequence of Chr VII (409354)	3-R
RHY105F	TCAATAATGGGACACATAAGCGA	gRNA for LHZ900 CRISPR plasmid	
RHY105R	AACTCGCTTATGTGTCCCATTAT	gRNA for LHZ900 CRISPR plasmid	
RHY105-up-F	ACTCTCTTGTGTAATCCATGGGATC	PCR for amplifying the upstream sequence of Chr I (1673871)	2-F
RHY105-up-R	CTTAGCGTGTGAATTTATTTAGCACCATAACTTCGTATAATGTACATTATACGAAGTTATATCGCTTATGTGTCCCATTATTTG	PCR for amplifying the upstream sequence of Chr I (1673871)	
RHY105-down-F	GGTGCTAAATAAATTCACACGCTAAG	PCR for amplifying the downstream sequence of Chr I (1673871)	
RHY105-down-R	ATGTATGGTGCGAATTTTATTCACC	PCR for amplifying the downstream sequence of Chr I (1673871)	2-R
RHY106F	TCAAGGGTTAGGTTTGAATGGTA	gRNA for LHZ901 CRISPR plasmid	
RHY106R	AACTACCATTCAAACCTAACCCT	gRNA for LHZ901 CRISPR plasmid	
RHY106-up-F	GCTTGGTTCCAAGATTTGACTTTAG	PCR for amplifying the upstream sequence of Chr II (896924)	10-F
RHY106-up-R	GAAGAGGGTTAGGTTTGAATGGTACATAACTTCGTATAATGTACATTATACGAAGTTATGGGGGTATGTTCAAAGAACAAAAAT	PCR for amplifying the upstream sequence of Chr II (896924)	
RHY106-down-F	GTACCATTCAAACCTAACCCTCTTC	PCR for amplifying the downstream sequence of Chr II (896924)	
RHY106-down-R	ATGATCGCTGGTTTGAAATCAATAC	PCR for amplifying the downstream sequence of Chr II (896924)	10-R
RHY107F	TCA GAATCATGGGCTTTTTGTAA	gRNA for LHZ902 CRISPR plasmid	
RHY107R	AAC TTACAAAAAGCCCATGATTC	gRNA for LHZ902 CRISPR plasmid	
RHY107-up-F	ATTGACAAGGCAACTAGGTAACACG	PCR for amplifying the upstream sequence of Chr I (1451427)	9-F
RHY107-up-R	TAGGAAAATCATTAATAATATCCTGCCATAACTTCGTATAATGTACATTATACGAAGTTATCTTACAAAAAGCCCATGATTCG	PCR for amplifying the upstream sequence of Chr I (1451427)	
RHY107-down-F	GGCAGGATATTATTAATGATTTTCCTA	PCR for amplifying the downstream sequence of Chr I (1451427)	
RHY107-down-R	GCGAAAGAAATTAAAAACAACAGAAAC	PCR for amplifying the downstream sequence of Chr I (1451427)	9-R
RHY108F	TCA CCGATTCTCTCCCCGCATCT	gRNA for LHZ903 CRISPR plasmid	
RHY108R	AAC AGATGCGGGGAGAGAATCGG	gRNA for LHZ903 CRISPR plasmid	
RHY108-up-F	ACTACTCGCACTCCACCATAATCC	PCR for amplifying the upstream sequence of Chr III (326046)	11-F
RHY108-up-R	TAAAAACGTTATTCTTTTAACCAGAGCCATAACTTCGTATAATGTACATTATACGAAGTTATGAGATGCGGGGAGAGAATCGG	PCR for amplifying the upstream sequence of Chr III (326046)	
RHY108-down-F	GGCTCTGGTTAAAAGAATAACGTTTTTA	PCR for amplifying the downstream sequence of Chr III (326046)	
RHY108-down-R	AGGTGTCTAGCAAATTGAACGAATC	PCR for amplifying the downstream sequence of Chr III (326046)	11-R
RHY109F	TCA GGTAATTATTTTCTTTCTAC	gRNA for LHZ904 CRISPR plasmid	
RHY109R	AAC GTAGAAAGAAAATAATTACC	gRNA for LHZ904 CRISPR plasmid	
RHY109-up-F	GCTATTGTAGTTGGGGGAAGTGG	PCR for amplifying the upstream sequence of Chr IV (704908)	12-F
RHY109-up-R	TTTACAACCTATAAACTATATCCATAACTTCGTATAATGTACATTATACGAAGTTATGGATATAGTTTATAGGTTGTAAAACTCTTTC	PCR for amplifying the upstream sequence of Chr IV (704908)	
RHY109-down-F	TGTAGAAAGAAAATAATTACCACTATTTATTTC	PCR for amplifying the downstream sequence of Chr IV (704908)	
RHY109-down-R	CGACTCTCTTGGTTTACAAGCTGAC	PCR for amplifying the downstream sequence of Chr IV (704908)	12-R
RHY110F	TCA GCACGGAGCAGATCTAGTTT	gRNA for LHZ905 CRISPR plasmid	
RHY110R	AAC AAACTAGATCTGCTCCGTGC	gRNA for LHZ905 CRISPR plasmid	
RHY110-up-F	TCTTGATGAACAGATGGTATGTTGC	PCR for amplifying the upstream sequence of Chr V (346042)	7-F
RHY110-up-R	GCAGAAGTCCTTATAATATATGTTGCCATAACTTCGTATAATGTACATTATACGAAGTTATTAAACTAGATCTGCTCCGTGCCAG	PCR for amplifying the upstream sequence of Chr V (346042)	
RHY110-down-F	GGCAACATATATTATAAGGACTTCTGC	PCR for amplifying the downstream sequence of Chr V (346042)	
RHY110-down-R	GGCAAAGCGATATAAAAAGAGATGA	PCR for amplifying the downstream sequence of Chr V (346042)	7-R

RHY111F	TCA CTTTACTTATGATGATAGGA	gRNA for LHZ906 CRISPR plasmid	
RHY111R	AAC TCCTATCATCATAAGTAAAG	gRNA for LHZ906 CRISPR plasmid	
RHY111-up-F	ACTTGCGGATCTTCTCTGATGTATC	PCR for amplifying the upstream sequence of Chr VI (1069906)	13-F
RHY111-up-R	TGTTCTTTACTTATGATGATAGGAAATAACTTCG TATAATGTACATTATACGAAGTTATGGACATTTT TTTTAAGCCTTGATAGC	PCR for amplifying the upstream sequence of Chr VI (1069906)	
RHY111-down-F	TTCCTATCATCATAAGTAAAGAACATTTTTTTC	PCR for amplifying the downstream sequence of Chr VI (1069906)	
RHY111-down-R	AAGAAGAGCAGGAGGATCTGGAG	PCR for amplifying the downstream sequence of Chr VI (1069906)	13-R
RHY112F	TCA AGCATAAGTCGTGATATTCC	gRNA for LHZ907 CRISPR plasmid	
RHY112R	AAC GGAATATCACGACTTATGCT	gRNA for LHZ907 CRISPR plasmid	
RHY112-up-F	GGGCAAGCGAAGTGTTACTGAAG	PCR for amplifying the upstream sequence of Chr III (1219332)	5-F
RHY112-up-R	TGGAATATCACGACTTATGCTGGT	PCR for amplifying the upstream sequence of Chr III (1219332)	
RHY112-down-F	CCAGCATAAGTCGTGATATTCCAATAACTTCGT ATAATGTACATTATACGAAGTTATGGCGAAATA AACAACAACTTCCA	PCR for amplifying the downstream sequence of Chr III (1219332)	
RHY112-down-R	CTATATACTGGACGGATGCATGGAG	PCR for amplifying the downstream sequence of Chr III (1219332)	5-R
RHY113F	TCA TGAGAACCAGAAAGATTAGA	gRNA for LHZ908 CRISPR plasmid	
RHY113R	AAC TCTAATCTTTCTGGTTCTCA	gRNA for LHZ908 CRISPR plasmid	
RHY113-up-F	CTGGAAATACGTCTTGCAGTAAACA	PCR for amplifying the upstream sequence of Chr IV (1172245)	8-F
RHY113-up-R	ATCACTCACTCAAATATCCCTAGCCATAACTTCG TATAATGTACATTATACGAAGTTATTTCTAATCT TTCTGGTTCTCACTTCG	PCR for amplifying the upstream sequence of Chr IV (1172245)	
RHY113-down-F	GGCTAGGGATATTTGAGTGAGTGAT	PCR for amplifying the downstream sequence of Chr IV (1172245)	
RHY113-down-R	CTGCTGAAAACTTCAGAGCTTTATG	PCR for amplifying the downstream sequence of Chr IV (1172245)	8-R
RHY114F	TCA GTCCGTCGCACTACTGTATG	gRNA for LHZ909 CRISPR plasmid	
RHY114R	AAC CATACAGTAGTGCGACGGAC	gRNA for LHZ909 CRISPR plasmid	
RHY114-up-F	GTTGTCTTCTAGTGTGACGTCGATG	PCR for amplifying the upstream sequence of Chr V (756338)	16-F
RHY114-up-R	GTTATTATTATGTATTTGGGCAGTCCATAACTTC GTATAATGTACATTATACGAAGTTATTCATACA GTAGTGCGACGGACTCTTG	PCR for amplifying the upstream sequence of Chr V (756338)	
RHY114-down-F	GGACTGCCCAAATACATAATAATAACA	PCR for amplifying the downstream sequence of Chr V (756338)	
RHY114-down-R	ATTTGCGGTATTATTTGAGGATCTC	PCR for amplifying the downstream sequence of Chr V (756338)	16-R
RHY115F	TCA AAGGATGATAATAGTGCATT	gRNA for LHZ910 CRISPR plasmid	
RHY115R	AAC AATGCACTATTATCATCCTT	gRNA for LHZ910 CRISPR plasmid	
RHY115-up-F	TCTACAGCATACTCCCTATCTGCAG	PCR for amplifying the upstream sequence of Chr VI (753935)	6-F
RHY115-up-R	TAATGCACTATTATCATCCTTAAATTCTAAT	PCR for amplifying the upstream sequence of Chr VI (753935)	
RHY115-down-F	ATTTAAGGATGATAATAGTGCATTAATAACTTC GTATAATGTACATTATACGAAGTTATGGCTACA CTACAACTACGACACCTATC	PCR for amplifying the downstream sequence of Chr VI (753935)	
RHY115-down-R	GAAATGGTTTATGAAATTTCCCAAG	PCR for amplifying the downstream sequence of Chr VI (753935)	6-R
RHY116F	TCA GATCTATTATTATAATTATG	gRNA for LHZ911 CRISPR plasmid	
RHY116R	AAC CATAATTATAATAATAGATC	gRNA for LHZ911 CRISPR plasmid	
RHY116-up-F	TTTTCCAGAGGCATTATACGAAAAG	PCR for amplifying the upstream sequence of Chr VII (689479)	14-F
RHY116-up-R	TAATGATCTATTATTATAATTATGAATAACTTCG TATAATGTACATTATACGAAGTTATGGCTTTAAT TGTTTTCCTCGTTATC	PCR for amplifying the upstream sequence of Chr VII (689479)	
RHY116-down-F	TCATAATTATAATAATAGATCATTAATTAAACTG AC	PCR for amplifying the downstream sequence of Chr VII (689479)	
RHY116-down-R	AATTTGGATGATGATATACCTCCTCACAG	PCR for amplifying the downstream sequence of Chr VII (689479)	14-R
RHY117F	TCA AGCGTATATATTCTCGCTTC	gRNA for LHZ912 CRISPR plasmid	
RHY117R	AAC GAAGCGAGAATATATACGCT	gRNA for LHZ912 CRISPR plasmid	
RHY117-up-F	ATGGTTGTTAAAGTTTTGAAGCAAAC	PCR for amplifying the upstream sequence of Chr II (717538)	4-F
RHY117-up-R	GTGAGGAGGGTAGTGAGCTTTTCCATAACTTCG TATAATGTACATTATACGAAGTTATAGAAGCGA GAATATATACGCTTATAACTTTTAG	PCR for amplifying the upstream sequence of Chr II (717538)	
RHY117-down-F	GGAAAAGCTCACTACCCTCCTCAC	PCR for amplifying the downstream sequence of Chr II (717538)	
RHY117-down-R	CTGTACAGAGACATAGTAACCAGGGATTC	PCR for amplifying the downstream sequence of Chr II (717538)	4-R
RHY118F	TCA TGATTAAAGACAGAAATGAT	gRNA for LHZ913 CRISPR plasmid	
RHY118R	AAC ATCATTTCTGTCTTTAATCA	gRNA for LHZ913 CRISPR plasmid	
RHY118-up-F	TCTTCAACTTATGTGCCACAAGATC	PCR for amplifying the upstream sequence of Chr VIII (610289)	1-F

RHY118-up-R	TATAGGTTGTAATACGTTCATGACTATAACTTCG TATAATGTACATTATACGAAGTTATGGTTCAAA AACTAATTTCTCAATTCAG	PCR for amplifying the upstream sequence of Chr VIII (610289)	
RHY118-down-F	AGTCATGAACGTATTACAACCTATATCC	PCR for amplifying the downstream sequence of Chr VIII (610289)	
RHY118-down-R	AGACTTCTTCTGGTAGAAACGGAAG	PCR for amplifying the downstream sequence of Chr VIII (610289)	1-R
