# Supplementary material for: Establishment of a Cre-loxP System Based on a Leaky LAC4 Promoter and an Unstable panARS Element in Kluyveromyces marxianus
Source: Microorganisms. 2022 Jun 17;10(6):1240. doi: 10.3390/microorganisms10061240 (PMC9227491; doi:10.3390/microorganisms10061240)
Supplement: Supplementary file 1 [file microorganisms-10-01240-s001.zip › Supplementary File S4.pdf]

Supplementary file 4

LOCUS LHZ881 6496 bp DNA circular 30-NOV-2021
SOURCE
ORGANISM
COMMENT This file is created by Vector NTI
http://www.invitrogen.com/
COMMENT ORIGDB|GenBank
COMMENT VNTDATE|-12027597|
COMMENT VNTDBDATE|-14155143|
COMMENT LSOWNER|
COMMENT VNTNAME|LHZ881|
COMMENT VNTAUTHORNAME|Demo User|
COMMENT VNTOAUTHORNAME|Yao Yu|
FEATURES Location/Qualifiers
terminator 2189..2378
/vntifkey=""43""
/label=ADH1\Terminator
CDS complement(5169..6029)
/vntifkey=""4""
/label=Lactamase-AmpC
promoter 2416..2794
/vntifkey=""29""
/label=TEF\Promoter
CDS 2795..3823
/vntifkey=""4""
/label=HphMX4
terminator 3824..4058
/vntifkey=""43""
/label=TEF\terminator
promoter 716..2162
/vntifkey=""30""
/label=ADH1\promoter
CDS 25..703
/vntifkey=""4""
/label=kmARS1
BASE COUNT 1742 a 1496 c 1496 g 1762 t
ORIGIN
1 gaacgcggcc gccagctgaa gcttatcgat tgaagttttg tccaactatc cactatggat
61 atgcgttttg ttgattaacc ttaaataaca cgtatttcgc attttccaaa agcctttttt
121 cataactaca aactaactat ttttgtttat tttacatcag taaattatgc gcagaaaata
181 tgtaaggcta tatactcaat atagtggaag agcctctggc tacttaatct gggttcatat
241 attctgtcag tggtatagta aatttaaata gtgaatttgg gcgcatgtag agagatcttt

301 tgattaataa cccagtattt gattcttgaa tgttatttgt ctcttattaa gtatttcagt
361 ttgatttata tattttgaaa gtaattgttg ctttaccttc caaacaataa aaaaatataa
421 aaaaatgtaa aaaatataat aaatattaaa taaaatacta cttgtttgaa aatcagagaa
481 aatccaccaa aatatcaatc atttcaagga tttccgaacc aagttcgaga tagtccttta
541 aggtcagtaa aattcagttg cacgtataac agatattttt cattttgttc caatttaaaa
601 gtcccccatt tttaaaatta ttgaaaataa aaattaaaaa attaaaagga atctctctat
661 gtcactttaa aataaataaa ttgaaaatga tatatcgtta aaaccgcggg tcgacgaaga
721 aatgatggta aatgaaatag gaaatcaagg agcatgaagg caaaagacaa atataagggt
781 cgaacgaaaa ataaagtgaa aagtgttgat atgatgtatt tggctttgcg gcgccgaaaa
841 aacgagttta cgcaattgca caatcatgct gactctgtgg cggacccgcg ctcttgccgg
901 cccggcgata acgctgggcg tgaggctgtg cccggcggag ttttttgcgc ctgcattttc
961 caaggtttac cctgcgctaa ggggcgagat tggagaagca ataagaatgc cggttggggt
1021 tgcgatgatg acgaccacga caactggtgt cattatttaa gttgccgaaa gaacctgagt
1081 gcatttgcaa catgagtata ctagaagaat gagccaagac ttgcgagacg cgagtttgcc
1141 ggtggtgcga acaatagagc gaccatgacc ttgaaggtga gacgcgcata accgctagag
1201 tactttgaag aggaaacagc aatagggttg ctaccagtat aaatagacag gtacatacaa
1261 cactggaaat ggttgtctgt ttgagtacgc tttcaattca tttgggtgtg cactttatta
1321 tgttacaata tggaagggaa ctttacactt ctcctatgca catatattaa ttaaagtcca
1381 atgctagtag agaagggggg taacacccct ccgcgctctt ttccgatttt tttctaaacc
1441 gtggaatatt tcggatatcc ttttgttgtt tccgggtgta caatatggac ttcctctttt
1501 ctggcaacca aacccataca tcgggattcc tataatacct tcgttggtct ccctaacatg
1561 taggtggcgg aggggagata tacaatagaa cagataccag acaagacata atgggctaaa
1621 caagactaca ccaattacac tgcctcattg atggtggtac ataacgaact aatactgtag
1681 ccctagactt gatagccatc atcatatcga agtttcacta cccttttttcc atttgccatc
1741 tattgaagta ataataggcg catgcaactt cttttctttt tttttctttt ctctctcccc
1801 cgttgttgtc tcaccatatc cgcaatgaca aaaaaaatga tggaagacac taaaggaaaa
1861 aattaacgac aaagacagca ccaacagatg tcgttgttcc agagctgatg aggggtatct
1921 cgaagcacac gaaacttttt ccttccttca ttcacgcaca ctactctcta atgagcaacg
1981 gtatacggcc ttccttccag ttacttgaat ttgaaataaa aaaaagtttg ctgtcttgct
2041 atcaagtata aatagacctg caattattaa tcttttgttt cctcgtcatt gttctcgttc
2101 cctttcttcc ttgtttcttt ttctgcacaa tatttcaagc tataccaagc atacaatcaa
2161 ctcccgggta gcggtaccgg tactcgaggc gaatttctta tgatttatga tttttattat
2221 taaataagtt ataaaaaaaa taagtgtata caaattttaa agtgactctt aggttttaaa
2281 acgaaaattc ttattcttga gtaactcttt cctgtaggtc aggttgcttt ctcaggtata
2341 gtatgaggtc gctcttattg accacacctc taccggcaga tccgctaggg ataacagggt
2401 aatatagatc tgtttagctt gccttgtccc cgccgggtca cccggccagc gacatggagg
2461 cccagaatac cctccttgac agtcttgacg tgcgcagctc aggggcatga tgtgactgtc
2521 gcccgtacat ttagcccata catccccatg tataatcatt tgcatccata cattttgatg
2581 gccgcacggc gcgaagcaaa aattacggct cctcgctgca gacctgcgag cagggaaacg
2641 ctcccctcac agacgcgttg aattgtcccc acgccgcgcc cctgtagaga aatataaaag
2701 gttaggattt gccactgagg ttcttctttc atatacttcc ttttaaaatc ttgctaggat
2761 acagttctca catcacatcc gaacataaac aaccatgggt aaaaagcctg aactcaccgc
2821 gacgtctgtc gagaagtttc tgatcgaaaa gttcgacagc gtctccgacc tgatgcagct
2881 ctcggagggc gaagaatctc gtgctttcag cttcgatgta ggagggcgtg gatatgtcct

2941 gcgggtaaat agctgcgccg atggtttcta caaagatcgt tatgtttatc ggcactttgc
3001 atcggccgcg ctcccgattc cggaagtgct tgacattggg gaattcagcg agagcctgac
3061 ctattgcatc tcccgccgtg cacagggtgt cacgttgcaa gacctgcctg aaaccgaact
3121 gcccgctgtt ctgcagccgg tcgcggaggc catggatgcg atcgctgcgg ccgatcttag
3181 ccagacgagc gggttcggcc cattcggacc gcaaggaatc ggtcaataca ctacatggcg
3241 tgatttcata tgcgcgattg ctgatcccca tgtgtatcac tggcaaactg tgatggacga
3301 caccgtcagt gcgtccgtcg cgcaggctct cgatgagctg atgctttggg ccgaggactg
3361 ccccgaagtc cggcacctcg tgcacgcgga tttcggctcc aacaatgtcc tgacggacaa
3421 tggccgcata acagcggtca ttgactggag cgaggcgatg ttcggggatt cccaatacga
3481 ggtcgccaac atcttcttct ggaggccgtg gttggcttgt atggagcagc agacgcgcta
3541 cttcgagcgg aggcatccgg agcttgcagg atcgccgcgg ctccgggcgt atatgctccg
3601 cattggtctt gaccaactct atcagagctt ggttgacggc aatttcgatg atgcagcttg
3661 ggcgcagggt cgatgcgacg caatcgtccg atccggagcc gggactgtcg ggcgtacaca
3721 aatcgcccgc agaagcgcgg ccgtctggac cgatggctgt gtagaagtac tcgccgatag
3781 tggaaaccga cgccccagca ctcgtccgag ggcaaaggaa taatcagtac tgacaataaa
3841 aagattcttg ttttcaagaa cttgtcattt gtatagtttt tttatattgt agttgttcta
3901 ttttaatcaa atgttagcgt gatttatatt ttttttcgcc tcgacatcat ctgcccagat
3961 gcgaagttaa gtgcgcagaa agtaatatca tgcgtcaatc gtatgtgaat gctggtcgct
4021 atactgctgt cgattcgata ctaacgccgc catccagtgt cgaaaacgag ctcgaattca
4081 tcgatgatat cagatccact agtggcctat gcggccgcgg atctgccggt ctccctatag
4141 tgagtcgtat taatttcgat aagccaggtt aacctgcatt aatgaatcgg ccaacgcgcg
4201 gggagaggcg gtttgcgtat tgggcgctct tccgcttcct cgctcactga ctcgctgcgc
4261 tcggtcgttc ggctgcggcg agcggtatca gctcactcaa aggcggtaat acggttatcc
4321 acagaatcag gggataacgc aggaaagaac atgtgagcaa aaggccagca aaaggccagg
4381 aaccgtaaaa aggccgcgtt gctggcgttt ttccataggc tccgcccccc tgacgagcat
4441 cacaaaaatc gacgctcaag tcagaggtgg cgaaacccga caggactata aagataccag
4501 gcgtttcccc ctggaagctc cctcgtgcgc tctcctgttc cgaccctgcc gcttaccgga
4561 tacctgtccg cctttctccc ttcgggaagc gtggcgcttt ctcaatgctc acgctgtagg
4621 tatctcagtt cggtgtaggt cgttcgctcc aagctgggct gtgtgcacga accccccgtt
4681 cagcccgacc gctgcgcctt atccggtaac tatcgtcttg agtccaaccc ggtaagacac
4741 gacttatcgc cactggcagc agccactggt aacaggatta gcagagcgag gtatgtaggc
4801 ggtgctacag agttcttgaa gtggtggcct aactacggct acactagaag gacagtattt
4861 ggtatctgcg ctctgctgaa gccagttacc ttcggaaaaa gagttggtag ctcttgatcc
4921 ggcaaacaaa ccaccgctgg tagcggtggt ttttttgttt gcaagcagca gattacgcgc
4981 agaaaaaaag gatctcaaga agatcctttg atcttttcta cggggtctga cgctcagtgg
5041 aacgaaaact cacgttaagg gattttggtc atgagattat caaaaaggat cttcacctag
5101 atccttttaa attaaaaatg aagttttaaa tcaatctaaa gtatatatga gtaaacttgg
5161 tctgacagtt accaatgctt aatcagtgag gcacctatct cagcgatctg tctatttcgt
5221 tcatccatag ttgcctgact ccccgtcgtg tagataacta cgatacggga gggcttacca
5281 tctggcccca gtgctgcaat gataccgcga gacccacgct caccggctcc agatttatca
5341 gcaataaacc agccagccgg aagggccgag cgcagaagtg gtcctgcaac tttatccgcc
5401 tccatccagt ctattaattg ttgccgggaa gctagagtaa gtagttcgcc agttaatagt
5461 ttgcgcaacg ttgttgccat tgctacaggc atcgtggtgt cacgctcgtc gtttggtatg
5521 gcttcattca gctccggttc ccaacgatca aggcgagtta catgatcccc catgttgtgc

5581 aaaaaagcgg ttagctcctt cggtcctccg atcgttgtca gaagtaagtt ggccgcagtg
5641 ttatcactca tggttatggc agcactgcat aattctctta ctgtcatgcc atccgtaaga
5701 tgcttttctg tgactggtga gtactcaacc aagtcattct gagaatagtg tatgcggcga
5761 ccgagttgct cttgcccggc gtcaatacgg gataataccg cgccacatag cagaacttta
5821 aaagtgctca tcattggaaa acgttcttcg gggcgaaaac tctcaaggat cttaccgctg
5881 ttgagatcca gttcgatgta acccactcgt gcacccaact gatcttcagc atcttttact
5941 ttcaccagcg tttctgggtg agcaaaaaca ggaaggcaaa atgccgcaaa aaagggaata
6001 agggcgacac ggaaatgttg aatactcata ctcttccttt ttcaatatta ttgaagcatt
6061 tatcagggtt attgtctcat gagcggatac atatttgaat gtatttagaa aaataaacaa
6121 ataggggttc cgcgcacatt tccccgaaaa gtgccacctg acgtctaaga aaccattatt
6181 atcatgacat taacctataa aaataggcgt atcacgaggc cctttcgtct cgcgcgtttc
6241 ggtgatgacg gtgaaaacct ctgacacatg cagctcccgg agacggtcac agcttgtctg
6301 taagcggatg ccgggagcag acaagcccgt cagggcgcgt cagcgggtgt tggcgggtgt
6361 cggggctggc ttaactatgc ggcatcagag cagattgtac tgagagtgca ccatatggac
6421 atattgtcgt tagaacgcgg ctacaattaa tacataacct tatgtatcat acacatacga
6481 tttaggtgac actata

//
