# Supplementary material for: Establishment of a Cre-loxP System Based on a Leaky LAC4 Promoter and an Unstable panARS Element in Kluyveromyces marxianus
Source: Microorganisms. 2022 Jun 17;10(6):1240. doi: 10.3390/microorganisms10061240 (PMC9227491; doi:10.3390/microorganisms10061240)
Supplement: Supplementary file 1 [file microorganisms-10-01240-s001.zip › Supplementary File S5.pdf]

Supplementary file 5

```
LOCUS       LHZ882        6702 bp    DNA     circular
19-JUL-2021
SOURCE
  ORGANISM
COMMENT     This file is created by Vector NTI
            http://www.invitrogen.com/
COMMENT     ORIGDB|GenBank
COMMENT     VNTDATE|-12027597|
COMMENT     VNTDBDATE|-13961859|
COMMENT     LSOWNER|
COMMENT     VNTNAME|LHZ882|
COMMENT     VNTAUTHORNAME|Demo User|
COMMENT     VNTOAUTHORNAME|Yao Yu|
FEATURES             Location/Qualifiers
     terminator      2395..2584
                     /vntifkey=""43""
                     /label=ADH1\Terminator
     CDS             complement(5375..6235)
                     /vntifkey=""4""
                     /label=Lactamase-AmpC
     promoter        2622..3000
                     /vntifkey=""29""
                     /label=TEF\Promoter
     CDS             3001..4029
                     /vntifkey=""4""
                     /label=HphMX4
     terminator      4030..4264
                     /vntifkey=""43""
                     /label=TEF\terminator
     promoter        922..2368
                     /vntifkey=""30""
                     /label=ADH1\promoter
     centromere      710..909
                     /vntifkey=""5""
                     /label=cen5
     CDS             25..703
                     /vntifkey=""4""
                     /label=kmARS1
BASE COUNT    1821 a     1509 c     1514 g     1858 t
ORIGIN
        1 gaacgcggcc gccagctgaa gcttatcgat tgaagttttg tccaactatc cactatggat
       61 atgcgttttg ttgattaacc ttaaataaca cgtatttcgc attttccaaa agcctttttt
```

121 cataactaca aactaactat ttttgtttat tttacatcag taaattatgc gcagaaaata
181 tgtaaggcta tatactcaat atagtggaag agcctctggc tacttaatct gggttcatat
241 attctgtcag tggtatagta aatttaaata gtgaatttgg gcgcatgtag agagatcttt
301 tgattaataa cccagtattt gattcttgaa tgttatttgt ctcttattaa gtatttcagt
361 ttgatttata tattttgaaa gtaattgttg ctttaccttc caaacaataa aaaaatataa
421 aaaaatgtaa aaaatataat aaatattaaa taaaatacta cttgtttgaa aatcagagaa
481 aatccaccaa aatatcaatc atttcaagga tttccgaacc aagttcgaga tagtccttta
541 aggtcagtaa aattcagttg cacgtataac agatattttt cattttgttc caatttaaaa
601 gtcccccatt tttaaaatta ttgaaaataa aaattaaaaa attaaaagga atctctctat
661 gtcactttaa aataaataaa ttgaaaatga tatatcgtta aaaccgcggg tcacgtgtat
721 acaaattatt aattttaaaa ctatataatt tattattttt ttattttgaa gtttagagta
781 attttagtag tattttatat tttaaataaa tatgctttaa atttttactt aatattttat
841 tatttttaaa tacaacgttt ttatttaaaa caaaattata agttaaaaag ttgttccgaa
901 agtaaaatag gatccgtcga cgaagaaatg atggtaaatg aaataggaaa tcaaggagca
961 tgaaggcaaa agacaaatat aagggtcgaa cgaaaaataa agtgaaaagt gttgatatga
1021 tgtatttggc tttgcggcgc cgaaaaaacg agtttacgca attgcacaat catgctgact
1081 ctgtggcgga cccgcgctct tgccggcccg gcgataacgc tgggcgtgag gctgtgcccg
1141 gcggagtttt ttgcgcctgc attttccaag gtttaccctg cgctaagggg cgagattgga
1201 gaagcaataa gaatgccggt tggggttgcg atgatgacga ccacgacaac tggtgtcatt
1261 atttaagttg ccgaaagaac ctgagtgcat ttgcaacatg agtatactag aagaatgagc
1321 caagacttgc gagacgcgag tttgccggtg gtgcgaacaa tagagcgacc atgaccttga
1381 aggtgagacg cgcataaccg ctagagtact ttgaagagga aacagcaata gggttgctac
1441 cagtataaat agacaggtac atacaacact ggaaatggtt gtctgtttga gtacgctttc
1501 aattcatttg ggtgtgcact ttattatgtt acaatatgga agggaacttt acacttctcc
1561 tatgcacata tattaattaa agtccaatgc tagtagagaa ggggggtaac acccctccgc
1621 gctcttttcc gattttttc taaaccgtgg aatatttcgg atatcctttt gttgtttccg
1681 ggtgtacaat atggacttcc tcttttctgg caaccaaacc catacatcgg gattcctata
1741 ataccttcgt tggtctccct aacatgtagg tggcggaggg gagatataca atagaacaga
1801 taccagacaa gacataatgg gctaaacaag actacaccaa ttacactgcc tcattgatgg
1861 tggtacataa cgaactaata ctgtagccct agacttgata gccatcatca tatcgaagtt
1921 tcactaccct ttttccattt gccatctatt gaagtaataa taggcgcatg caacttcttt
1981 tctttttttt tcttttctct ctccccgtt gttgtctcac catatccgca atgacaaaaa
2041 aaatgatgga agacactaaa ggaaaaaatt aacgacaaag acagcaccaa cagatgtcgt
2101 tgttccagag ctgatgaggg gtatctcgaa gcacacgaaa ctttttcctt ccttcattca
2161 cgcacactac tctctaatga gcaacggtat acggccttcc ttccagttac ttgaatttga
2221 aataaaaaaa agtttgctgt cttgctatca agtataaata gacctgcaat tattaatctt
2281 ttgtttcctc gtcattgttc tcgttccctt tcttccttgt ttctttttct gcacaatatt
2341 tcaagctata ccaagcatac aatcaactcc cgggtagcgg taccggtact cgaggcgaat
2401 ttcttatgat ttatgatttt tattattaaa taagttataa aaaaaataag tgtatacaaa
2461 ttttaaagtg actcttaggt tttaaaacga aaattcttat tcttgagtaa ctctttcctg
2521 taggtcaggt tgctttctca ggtatagtat gaggtcgctc ttattgacca cacctctacc
2581 ggcagatccg ctagggataa cagggtaata tagatctgtt tagcttgcct tgtccccgcc
2641 gggtcacccg gccagcgaca tggaggccca gaataccctc cttgacagtc ttgacgtgcg
2701 cagctcaggg gcatgatgtg actgtcgccc gtacatttag cccatacatc cccatgtata

2761 atcatttgca tccatacatt ttgatggccg cacggcgcga agcaaaaatt acggctcctc
2821 gctgcagacc tgcgagcagg gaaacgctcc cctcacagac gcgttgaatt gtccccacgc
2881 cgcgcccctg tagagaaata taaaaggtta ggatttgcca ctgaggttct tctttcatat
2941 acttcctttt aaaatcttgc taggatacag ttctcacatc acatccgaac ataaacaacc
3001 atgggtaaaa agcctgaact caccgcgacg tctgtcgaga agtttctgat cgaaaagttc
3061 gacagcgtct ccgacctgat gcagctctcg gagggcgaag aatctcgtgc tttcagcttc
3121 gatgtaggag ggcgtggata tgtcctgcgg gtaaatagct gcgccgatgg tttctacaaa
3181 gatcgttatg tttatcggca ctttgcatcg gccgcgctcc cgattccgga agtgcttgac
3241 attggggaat tcagcgagag cctgacctat tgcatctccc gccgtgcaca gggtgtcacg
3301 ttgcaagacc tgcctgaaac cgaactgccc gctgttctgc agccggtcgc ggaggccatg
3361 gatgcgatcg ctgcggccga tcttagccag acgagcgggt tcggcccatt cggaccgcaa
3421 ggaatcggtc aatacactac atggcgtgat ttcatatgcg cgattgctga tccccatgtg
3481 tatcactggc aaactgtgat ggacgacacc gtcagtgcgt ccgtcgcgca ggctctcgat
3541 gagctgatgc tttgggccga ggactgcccc gaagtccggc acctcgtgca cgcggatttc
3601 ggctccaaca atgtcctgac ggacaatggc cgcataacag cggtcattga ctggagcgag
3661 gcgatgttcg gggattccca atacgaggtc gccaacatct tcttctggag gccgtggttg
3721 gcttgtatgg agcagcagac gcgctacttc gagcggaggc atccggagct tgcaggatcg
3781 ccgcggctcc gggcgtatat gctccgcatt ggtcttgacc aactctatca gagcttggtt
3841 gacggcaatt tcgatgatgc agcttgggcg cagggtcgat gcgacgcaat cgtccgatcc
3901 ggagccggga ctgtcgggcg tacacaaatc gcccgcagaa gcgcggccgt ctggaccgat
3961 ggctgtgtag aagtactcgc cgatagtgga aaccgacgcc ccagcactcg tccgagggca
4021 aaggaataat cagtactgac aataaaaaga ttcttgtttt caagaacttg tcatttgtat
4081 agttttttta tattgtagtt gttctatttt aatcaaatgt tagcgtgatt tatatttttt
4141 ttcgcctcga catcatctgc ccagatgcga agttaagtgc gcagaaagta atatcatgcg
4201 tcaatcgtat gtgaatgctg gtcgctatac tgctgtcgat tcgatactaa cgccgccatc
4261 cagtgtcgaa aacgagctcg aattcatcga tgatatcaga tccactagtg gcctatgcgg
4321 ccgcggatct gccggtctcc ctatagtgag tcgtattaat ttcgataagc caggttaacc
4381 tgcattaatg aatcggccaa cgcgcgggga gaggcggttt gcgtattggg cgctcttccg
4441 cttcctcgct cactgactcg ctgcgctcgg tcgttcggct gcggcgagcg gtatcagctc
4501 actcaaaggc ggtaatacgg ttatccacag aatcagggga taacgcagga aagaacatgt
4561 gagcaaaagg ccagcaaaag gccaggaacc gtaaaaaggc cgcgttgctg gcgtttttcc
4621 ataggctccg cccccctgac gagcatcaca aaaatcgacg ctcaagtcag aggtggcgaa
4681 acccgacagg actataaaga taccaggcgt ttccccctgg aagctccctc gtgcgctctc
4741 ctgttccgac cctgccgctt accggatacc tgtccgcctt tctcccttcg ggaagcgtgg
4801 cgctttctca atgctcacgc tgtaggtatc tcagttcggt gtaggtcgtt cgctccaagc
4861 tgggctgtgt gcacgaaccc cccgttcagc ccgaccgctg cgccttatcc ggtaactatc
4921 gtcttgagtc caacccggta agacacgact tatcgccact ggcagcagcc actggtaaca
4981 ggattagcag agcgaggtat gtaggcggtg ctacagagtt cttgaagtgg tggcctaact
5041 acggctacac tagaaggaca gtatttggta tctgcgctct gctgaagcca gttaccttcg
5101 gaaaaagagt tggtagctct tgatccggca aacaaaccac cgctggtagc ggtggttttt
5161 ttgtttgcaa gcagcagatt acgcgcagaa aaaaaggatc tcaagaagat cctttgatct
5221 tttctacggg gtctgacgct cagtggaacg aaaactcacg ttaagggatt ttggtcatga
5281 gattatcaaa aaggatcttc acctagatcc ttttaaatta aaaatgaagt tttaaatcaa
5341 tctaaagtat atatgagtaa acttggtctg acagttacca atgcttaatc agtgaggcac

5401 ctatctcagc gatctgtcta tttcgttcat ccatagttgc ctgactcccc gtcgtgtaga
5461 taactacgat acgggagggc ttaccatctg gccccagtgc tgcaatgata ccgcgagacc
5521 cacgctcacc ggctccagat ttatcagcaa taaaccagcc agccggaagg gccgagcgca
5581 gaagtggtcc tgcaacttta tccgcctcca tccagtctat taattgttgc cgggaagcta
5641 gagtaagtag ttcgccagtt aatagtttgc gcaacgttgt tgccattgct acaggcatcg
5701 tggtgtcacg ctcgtcgttt ggtatggctt cattcagctc cggttcccaa cgatcaaggc
5761 gagttacatg atcccccatg ttgtgcaaaa aagcggttag ctccttcggt cctccgatcg
5821 ttgtcagaag taagttggcc gcagtgttat cactcatggt tatggcagca ctgcataatt
5881 ctcttactgt catgccatcc gtaagatgct tttctgtgac tggtgagtac tcaaccaagt
5941 cattctgaga atagtgtatg cggcgaccga gttgctcttg cccggcgtca atacgggata
6001 ataccgcgcc acatagcaga actttaaaag tgctcatcat tggaaaacgt tcttcggggc
6061 gaaaactctc aaggatctta ccgctgttga gatccagttc gatgtaaccc actcgtgcac
6121 ccaactgatc ttcagcatct tttactttca ccagcgtttc tgggtgagca aaaacaggaa
6181 ggcaaaatgc cgcaaaaaag ggaataaggg cgacacggaa atgttgaata ctcatactct
6241 tcctttttca atattattga agcatttatc agggttattg tctcatgagc ggatacatat
6301 ttgaatgtat ttagaaaaat aaacaaatag gggttccgcg cacatttccc cgaaaagtgc
6361 cacctgacgt ctaagaaacc attattatca tgacattaac ctataaaaat aggcgtatca
6421 cgaggccctt tcgtctcgcg cgtttcggtg atgacggtga aaacctctga cacatgcagc
6481 tcccggagac ggtcacagct tgtctgtaag cggatgccgg gagcagacaa gcccgtcagg
6541 gcgcgtcagc gggtgttggc gggtgtcggg gctggcttaa ctatgcggca tcagagcaga
6601 ttgtactgag agtgcaccat atggacatat tgtcgttaga acgcggctac aattaataca
6661 taaccttatg tatcatacac atacgattta ggtgacacta ta

//
